# Supplementary material for: Development of a competency profile for professionals involved in infectious disease preparedness and response in the air transport public health sector
Source: PLoS One. 2020 May 21;15(5):e0233360. doi: 10.1371/journal.pone.0233360 (PMC7241746; doi:10.1371/journal.pone.0233360)
Supplement: S1 File — (DOC) [file pone.0233360.s001.doc]

**S1. Literature searches**

**Content**

1. Competency profiles
   1. Search strategy
   2. Full text screening & selection
2. Airport guidelines
   1. Search strategy
   2. Full text screening & selection
3. **Competency profiles**
   1. **search strategy**

**Search terms:**

| **Infectious disease** | **AND** | **competence** |
| --- | --- | --- |
| Infectious diseases |  | competency |
| Communicable disease |  | competencies |
| Communicable diseases |  |  |
| Emergency preparedness |  |  |
| Emergency response |  |  |
|  |  |  |
|  |  |  |

**Inclusion criteria:**

- Contains an overview of competencies
- The competencies are apply to infectious disease preparedness or control specifically, or as part of general public health, disaster, or emergency preparedness.

**Exclusion criteria:**

- The profile delivers competencies for a broader scope in which infectious disease control is not separately treated.

**Pubmed**

((((((((((infectious disease[MeSH Terms]) OR "infectious disease"[Title/Abstract]) OR "infectious diseases"[Title/Abstract]) OR "communicable disease"[Title/Abstract]) OR "communicable diseases"[Title/Abstract]) OR "emergency preparedness"[Title/Abstract]) OR "emergency reponse"[Title/Abstract])) AND ((((competence[Title]) OR competency[Title]) OR competencies[Title]) OR competence[MeSH])))

**Results published in the last 5 years: 33 results**

**Title/abstract Screening led to inclusion of 1 article:**

- Stoto MA, Savoia E, Nelson C, Piltch-Loeb R, Guicciardi S, Takacs J, et al. Development of a public health emergency preparedness competency model for European Union countries. Euro Surveill. 2018 Dec;23(49). doi: 10.2807/1560-7917.ES.2018.23.48.1700631.

**Google Scholar, period 2014 – 2019.**

Search: “Infectious disease control competency profile” 🡪 title/abstract screening: 4 inclusions

- Brusaferro S, Cookson B, Kalenic S, Cooper T, Fabry J, Gallagher R, et al. Training infection control and hospital hygiene professionals in Europe, 2010: Agreed core competencies among 33 European countries. Euro Surveill. 2014;19(49):45-54.
- Burnett E, Curran E, Loveday HP, Kiernan MA, Tannahill M. The outcome competency framework for practitioners in infection prevention and control: use of the outcome logic model for evaluation. Journal of Infection Prevention. 2013;15(1): https://doi.org/10.1177/1757177413512387.
- Emery RJ, Patlovich JS, King K, Lowe JJ, Rios J. Comparing the Established Competency Categories of the Biosafety and Infection Prevention Professions: A Possible Roadmap for Addressing Professional Development Training Needs for a New Era. Applied biosafety. 2016;21(2): doi.org/10.1177/1535676016651250.
- Summers L. Evaluating and implementing public health workforce development initiatives to improve competencies of infectious disease staff at a local health department. Master Essay, Unviversity of Pittsbugh

Search: “Infectious disease control competencies” 🡪 title/abstract screening: 3 new inclusions

- Southall HG, DeYoung SE, Harris CA. Lack of cultural competency in international aid responses: the ebola outbreak in Liberia. Public health. 2017: <https://doi.org/10.3389/fpubh.2017.00005>
- Kaye K, Anderson DJ, Cook E, Huang SS. Guidance for infection prevention and healthcare epidemiology programs: healthcare epidemiologist skills and competencies. Infection control & hospital epidemiology. 2015;36(4):369-80.
- Cloeren M, Gean C, Kesler D, Green-McKenzi J, Taylor M, Upfal M, et al. American College of Occupational and Environmental Medicine's Occupational and Environmental Medicine Competencies—2014: ACOEM OEM Competencies Task Force. Journal of occupational and environmental medicine. 2014;556(5):21-40.

Search: “Infectious disease preparedness competencies” 🡪 title/abstract screening: 7 new inclusions

- Jose MM, Dufrene C. Educational competencies and technologies for disaster preparedness in undergraduate nursing education: an integrative review. Nurse education today. 2014;34(4):543-51.
- Loke AY, Man Fung OW. Nurses’ Competencies in Disaster Nursing: Implications for Curriculum Development and Public Health. Int J Environ Res Public Health. 2014;11(3):3289-3303.
- Stoto MA, Savoia E, Nelson C, Piltch-Loeb R, Guicciardi S, Takacs J, et al. Development of a public health emergency preparedness competency model for European Union countries. Euro Surveill. 2018:23(49):1700631.
- Waller KM. Emergency preparedness competencies among nurses in Northwest Arkansas. University of Arkansas, Fayetteville. Masterthesis 2017.
- Tartari E, Allegranzi B, Ang B, Calleja N, Collignon P et al. Preparedness of institutions around the world for managing patients with Ebola virus disease: an infection control readiness checklist. Antimicrobial Resistance and Infection Control. 2015;4(22).
- Matlock TA. Emergency Preparedness Competencies Among Nurses in Northwest Arkansas. University of Arkansas, Fayetteville. Masterthesis 2017.
- Czabanowska K, Smith T, Konings KD, Sumskas L, Otok R, Bjegovic-Mikanovic V, Brand H. In search for a public health leadership competency framework to support leadership curriculum – consensus study. European Journal of Public Health. 2014;24(5):850-6.

Search: “Infectious disease preparedness competency profile” 🡪 title/abstract screening: 0 new inclusions

- -

Search: “Public health competency profile” 🡪 title/abstract screening: 4 new inclusions

- Bjegovic-Mikanovic V, Foldspang A, Jakubowski E, Muller-Nordhorn J, Otok R, Stjernberg L. Developing the public health framework. Eurohealth. 2015;21(1): 24-7.
- Foldspang A, Otok R, Czabanowska K, Bejgovic-Mikanovic V. Developing the public health workforce in Europe. The European Public Health Reference Framework (EPHRF): Its Council and online respository ASPHER. 2014.
- Neiworth LL, Allan S, D’ambrosio L, Coplen-Abrahamson M. Charting a course to competency: an approach to mapping public health core competencies to existing trainings. Health promotion practice. 2014;15(1): https://doi.org/10.1177%2F1524839913509274.
- Otok R, Foldspang A. Main competences and skills to perform Essential Public Health Operations, offered by Schools of Public Health in four European countries: a short pilot report. International Journal of Public Health. 2016;61:633-9.

Search: “Infectious disease competences” 🡪 title/abstract screening: 1 new inclusion

- Foldspang A. Towards a public health profession: the roles of essential public health operations and lists of competences. European Journal of Public Health. 2015;25(3):361-62.

Search: “Infectious disease preparedness competences” 🡪 title/abstract screening: 2 new inclusions

- Foldspang A, Birt CA, Otok R, editors. ASPHER’s European List of Core Competences for the Public Health Professional, 5^th^ edition. ASPHER. 2018.

Search: “Infectious disease control competences” 🡪 title/abstract screening: no new inclusions

- -

**ECDC publications (**[**https://www.ecdc.europa.eu/en/publications-data?f%5B0%5D=output_types%3A1244**](https://www.ecdc.europa.eu/en/publications-data?f%5B0%5D=output_types%3A1244)**): 2019 – 2014, sorted on relevance, screened until 20 irrelevant publications in a row:**

- Search: “Competence” 🡪 led to 5 inclusions
- Vaccine-preventable diseases and immunisation: core competencies. 2017. Available from: https://www.ecdc.europa.eu/sites/default/files/documents/VPD%20Competencies%20Training_Short_Technical%20report_final_0.pdf.
- Public health preparedness emergency preparedness: core competencies for EU Member States. 2017. Available from https://www.ecdc.europa.eu/sites/default/files/documents/public-health-emergency-preparedness-core-competencies-eu-member-states.pdf.
- Stoto MA, Savoia E, Nelson C, Piltch-Loeb R, Guicciardi S, Takacs J, et al. Development of a public health emergency preparedness competency model for European Union countries. Eurosurveillance 2018;23(49):1700631.
- Risk communication as a core public health competence in infectious disease management: development of the ECDC training curriculum and programme. Euro Surveill. 2016;21(14).
- Brusaferro S, Cookson B, Kalenic S, Cooper T, Fabry J, et al. Training infection control and hospital hygiene professionals in Europe, 2010: agreed competencies among 33 European countries. Euro Surveill. 2014;19(49).
- Search “Competency”: 🡪 Led to no new publications
- Search “Competencies”: 🡪 Led to no new publications

**WHO**: Any of the words: Competence, Competency, Competencies, in the title of the document

- No relevant results

**Conclusion**

The search for competency profiles and title/abstract screening led to 23 unique inclusions.

- 1. **Full text screening**

|  | **Need to haves: Inclusion criteria** | | | **In/exclusion** | **Nice to haves: applicability criteria** | | | **Selected** |
| --- | --- | --- | --- | --- | --- | --- | --- | --- |
| **Study included in ti/ab screening** | **Contains a competency profile** | **≥Graduate level / professionals in practice** | **Infectious diseases management** | **Based on the ‘need-to-haves’** | **Infectious disease management, full array** | **Peer-reviewed** | **Airport setting** | **Sum score of the ‘nice-to-haves’-critria** |
| Bjegovic-Mikanovic et al. 2015 | No | - | No | Excluded | - | - | - | - |
| Brusaferro S et al. 2014 | No | - | Yes | Excluded | - | - | - | - |
| Burnett E et al. 2013 | No | - | Yes | Excluded |  |  |  |  |
| Cloeren M et al. 2014 | Yes | Yes | Yes | Included | No | Yes | No | 1 |
| Czabanowska K et al. 2014 | Yes | Yes | No | Excluded | - | - | - | - |
| ECDC 2017 (vaccine-preventable diseases) | Yes | Yes | Yes | Included | No, only vaccine application | No | No | 0 |
| ECDC 2017 PH preparedness: core competencies for EU member states | Yes | Yes | Yes | Included | Yes | No | No | 1 |
| ECDC 2016 Risk communication | Yes | Yes | Yes | Included | No | Yes | No | 1 |
| Emery RJ et al. 2016 | Yes | Yes | No | Excluded | - | - | - | - |
| Foldspang A et al. 2014 | No | - | No | Excluded | - | - | - | - |
| Foldspang A 2015 | No | - | No | Excluded | - | - | - | - |
| Foldspang A et al. 2018 | Yes | Yes | Yes | Included | Yes | Yes | No | 2 |
| Jose MM et al. 2014 | Yes | No | Yes | Excluded | - | - | - | - |
| Kaye K et al. 2015 | Yes | Yes | Yes | Included | Partly | Yes | No | 1.5 |
| Loke AY et al. 2014 | Yes | Yes | No | Excluded | - | - | - | - |
| Matlock TA 2017 | Yes | Yes | No | Excluded | - | - | - | - |
| Neiworth LL et al. 2014 | No | - | No | Excluded | - | - | - | - |
| Otok R et al. 2016 | No (they use prior existing) | Yes | Yes | Excluded | - | - | - | - |
| Summers L 2017 | Yes | Yes | No | Excluded | - | - | - | - |
| Southall HG et al. 2017 | No | - | Yes | Excluded | - | - | - | - |
| Stoto M et al. 2018 | Yes | Yes | Yes | Included | Yes | Yes | No | 2 |
| Tartari E et al. 2015 | No, capacity check list | - | Yes | Excluded | - | - | - | - |
| Waller KM 2017 | Yes | Yes | No | Excluded | - | - | - | - |

- = not applicable

1. **Airport guidelines**
   1. **Search strategy**

**Search terms:**

| **infectious disease** | **AND** | **airport** |
| --- | --- | --- |
| infectious diseases |  | airports |
| communicable disease |  | air travel |
| communicable diseases |  | aerodromes |
| emergency preparedness |  | airdrome |
| emergency response |  | airdromes |
| Public health event |  | airfield |
| Public health events |  | airfields |
|  |  | flying field |
|  |  | flying fields |
|  |  | landing field |
|  |  | landing fields |
|  |  | air station |
|  |  | air stations |
|  |  | air terminal |
|  |  | air terminals |
|  |  | aviation |
|  |  | air transport |
|  |  | airplane |
|  |  | airplanes |
|  |  | Point of entry |
|  |  | Points of entry |

**Title/abstract screening criteria:**

**Inclusion criteria:**

- Describing the process of infectious disease management
- at airports, or in an air travel setting
- Generic approach, non-specific infectious diseases

**Exclusion criteria:**

- focused on a specific disease

**Pubmed**

Search:

(((((((((((((((((((((((((((((airport[Title]) OR airports[Title]) OR aerospace[Title]) OR airdrome[Title]) OR aerodrome[Title]) OR "air station"[Title]) OR "air stations"[Title]) OR "air terminal"[Title]) OR "air terminals"[Title]) OR aviation[Title]) OR airfield[Title]) OR airfields[Title]) OR frontier[Title]) OR frontiers[Title]) OR "air travel"[Title]) OR "air traveler"[Title]) OR "air travelers"[Title]) OR "air traveller"[Title]) OR "air travellers"[Title]) OR "air transport"[Title]) OR "air transportation"[Title]) OR airplane[Title]) OR airplanes[Title]) OR “point of entry”[Title]) OR “points of entry”[Title]) OR airports[MeSH Terms]) OR “Aerospace Medicine”[MeSH Terms]))) AND ((((((((((((((infectious disease[MeSH Terms]) OR "infectious disease"[Title/Abstract]) OR "infectious diseases"[Title/Abstract]) OR "communicable disease"[Title/Abstract]) OR "communicable diseases"[Title/Abstract]) OR "emergency preparedness"[Title/Abstract]) OR "emergency reponse"[Title/Abstract] OR “public health event”[Title/Abstract]) OR “public health events[Title/Abstract])))))

**2014 – March 2019 = 54 results**

**Title/Abstract screening led to inclusion of 6 for full text screening:**

- Marienau KJ. Communicable disease X (Ebola, MERS, TB, measles…)--coming soon to a neighborhood near you? Lessons learned about communicable disease and air travel. Travel Med Infect Dis. 2015 Jan-Feb;13(1):3-5.
- Huizer YL, Swaan CM, Leitmeyer KC, Timen A. Usefulness and applicability of infectious disease control measures in air travel: a review. Travel Med Infect Dis. 2015 Jan-Feb;13(1):19-30. doi: 10.1016/j.tmaid.2014.11.008. Epub 2014 Dec 4. Review.
- Glynn RW, Boland M; HSE Port Health Groups, Ireland. Ebola, Zika and the International Health Regulations - implications for Port Health Preparedness. Global Health. 2016 Nov 21;12(1):74.
- Ghassemi P, Pfefer TJ, Casamento JP, Simpson R, Wang Q. Best practices for standardized performance testing of infrared thermographs intended for fever screening. PLoS One. 2018 Sep 19;13(9):e0203302. doi: 10.1371/journal.pone.0203302.
- Findlater A, Bogoch II. Human Mobility and the Global Spread of Infectious Diseases: A Focus on Air Travel. Trends Parasitol. 2018 Sep;34(9):772-783. doi: 10.1016/j.pt.2018.07.004.
- Martin G, Boland M. Planning and preparing for public health threats at airports. Global Health. 2018 Mar 7;14(1):28. doi: 10.1186/s12992-018-0323-3.

**Google Scholar, period 2014 - 2019**

Search: “ Infectious disease management airports”. 🡪 led to 8 new inclusions:

- Martin G, Boland M. Planning and preparing for public health threats at airports. Globalization and health. 2018;14(28). Doi10.1186/s12992-018-0323-3.
- Cenciarelli O, Pietropaoli S, Frusteri L, Malizia A, Carestia M, D’amico F, et al. Biological emergency management: the case of Ebola 2014 and the air transportation involvement. J. Microb. Biochem Tech. 2014;6(6):247-53.
- Gold L, Balal E, HorakT, Cheu RL. Screening of Infectious Disease among International Travelers at Airports. Presented at Transportation Research Board 97^th^ Annual Meeting; 2018; Washington DC .
- Gosadi IM, BinSaeed M, Al-Hazmi AM, Fadl AA, Alharbi KH, Swarelzahab MM. Evaluation of applied public health emergency system at Prince Mohammed International Airport in Almedinah during Hajj season 2014: a qualitative case. BMC research. 2015;435(8): Doi:10.1186/s13104-015-1415-2.
- Harvey J, Ferrill J, Sundberg K…Contemporary threats of infectious disease pandemics and bioterrorism: an underestimated risk to aviation, border control and national security. Emerging Infectious Diseases. 2014;10(7):1258–1263.
- Glynn RW, Boland M. Ebola, Zika and the International Health Regulations–implications for port health preparedness. Globalization and health. 2016;12(74): Doi.10.1186/s12992-016-0173-9.
- Dausey DJ, Biedrzycki PA, Cook T, Teufel J, Vendeville M, Francis E. Planning and response to communicable disease on US domestic air flights. Epidemiology. 2016;6(225): doi:10.4172/2161-1165.1000225.
- Leussink S. Preventing Airborne Infectious Spreading: The role of airport level operations during epidemics. [Place]: TU Delft;2019.

“Infectious disease management air transport”. Title/abstract screening Included:

- Findlater A, Bogoch II. Human mobility and the global spread of infectious diseases: a focus on air travel. Trends Parasitol. 2018;34(9):772-83.

“Infectious disease management air travel”. Title/abstract screening Included:

- Nable jV, Tupe CL, Gehle BD, Brady WJ. In-flight medical emergencies during commercial travel. N Engl J Med. 2015;373(10):939-45.
- Naouri D, Lapostolle F, Rondet C, Ganansia O, Pateron D, Yordanov Y. Prevention of medical events during air travel: a narrative review. Am J Med. 2016;129(9):1000.e1-6.

“Infectious disease management point of entry”. Title/abstract screening Included:

- Memish ZA, Zumla A, Alhakeem RF, Assiri A, Turkestani A, Al Harby KD, et al. Hajj: infectious disease surveillance and control. Lancet. 2014;383(9934):2073-82.

“Public health event management airport”. Title/abstract screening Included:

- Chiu HH, Hsieh KW, Wu YC, Chou JH, Chang FY. Building core capacities at the designated points of entry according to the International Health Regulations 2005: a review of the progress and prospects in Taiwan. Glob Health Action. 2014;7:24516. Doi: 10.3402/gha.v7.24516.
- Boucard A. Preparing and responding to a public health event: Montreal Airport. icao.int [Year: unknown].
- Wilhelmi J. Airport Roles in Reducing Transmission of Communicable Diseases: Summary of a Workshop of the Airport Cooperative Research Program's 2018 Insight Event. Transportation Research Board Conference 2019.

“Public health event air transport”. Title/abstract screening Included:

- World Health Organization. Handbook for the management of public health events in air transport: updated with information on Ebola virus disease and Middle East respiratory syndrome. World Health Organization. 2016.

“Public health event air travel”. Title/abstract screening Included:

- -

“Public health event point of entry”. Title/abstract screening Included:

- World Health Organization. Coordinated public health surveillance between points of entry and national health surveillance systems: advising principles. World Health Organization. 2014.

“Emergency planning air transport”. Title/abstract screening Included:

“Emergency planning air travel”. Title/abstract screening Included:

“Emergency planning airport”. Title/abstract screening Included:

- Kraus J, Plos V, Vittek P. The New Approach to Airport Emergency Plans. International J Soc Behav Edu Bus Ind Eng. 2014;8(8).
- Kraus J, Vittek P, Plos V. Comprehensive emergency management for airport operator documentation. London: Production Management and Engineering Sciences; 2016. Doi: 10.1201/b19259-27.
- Alexander DE. Disaster and emergency planning for preparedness, response, and recovery. 2015. In: Oxoford Research Encyclopedia of Natural Hazard Science. Oxford University press: Oxford; 2015.

“Emergency planning point of entry”. Title/abstract screening Included:

- **-**

**ECDC publications: 2014 – 2019, sorted on relevance, screened until 20 irrelevant publications in a row:**

**“Airports”:**

- -

**“Air travel”:**

- Technical guidance on risk assessment guidelines for diseases transmitted on aircraft (RAGIDA). Part 2: Operational guidelines - Second edition

**“Air transport”:**

- Huizer YL, Swaan CM, Leitmeyer KC, Timen A. Usefulness and applicability of infectious disease control measures in air travel: a review. Travel Med Infect Dis. 2015 Jan-Feb;13(1):19-30. doi: 10.1016/j.tmaid.2014.11.008.

**“Point of entry”:**

- -

**WHO - EURO:** [**http://www.euro.who.int/en/search**](http://www.euro.who.int/en/search)

**“public health emergency point of entry” + snow ball referencing.** Title/abstract screening Included:

- Action plan to improve public health preparedness and response in the WHO European Region 2018–2023. 2019
- 🡪 WHO - Points of Entry:
  - International Health Regulations (2005). A guide for public health emergency contingency planning at designated points of entry (2012)
  - WHO Lyon Office IHR coordination
    - Publications:
      - International Health Regulations (2005). A guide for public health emergency contingency planning at designated points of entry (2012)
      - Coordinated public health surveillance between points of entry and national health surveillance systems: advising principles. World Health Organization - 2014 - apps.who.int
      - Handbook for the management of public health events in air transport: updated with information on Ebola virus disease and Middle East respiratory syndrome …World Health Organization - 2016
  - WHO - Ports, airports and ground crossings
- WHO Airports:
  - Guide to hygiene and sanitation in aviation. Third edition. Module 1: Water. Module 2: Cleaning and disinfection of facilities
  - 🡪 CAPSCA website
    - Management of public health emergencies in aviation — GRF Davos One Health Summit 2012
    - Guidelines for States Concerning the Management of Communicable Disease Posing a Serious Public Health Risk
    - Template For An Aviation Public Health Emergency Preparedness Plan
  - 🡪 AIRSAN website
    - AIRSAN. Contact Tracing – Collaboration between the Public Health and the Aviation Sector. May 2015.
    - AIRSAN - Remote risk assessment and management of communicable disease events on board an aircraft
- International health regulations (2005): a guide for public health emergency contingency planning at designated points of entry. World Health Organization - 2012 - iris.wpro.who.int

**WHO / Global: Any of the words: “**airport, airports, airfield, aviation”. Included via title/abstract screening:

- Minimizing health risks at airports , ports and ground crossings
- WHO | Guide to hygiene and sanitation in aviation
- WHO | Vector surveillance and control at ports, airports , and ground crossings
- WHO/Europe | Airports (see above)
- WHO/Europe | 13 countries in the European Region better equipped to assess their public health capacities at airports and ports

“Points of entry”

- International health regulations (2005): a guide for public health emergency contingency planning at designated points of entry. World Health Organization - 2012 - iris.wpro.who.int

**Conclusion**

The search for air travel guidelines and title/abstract screening led to 29 unique inclusions.

- 1. **full text screening**

|  | **Inclusion criteria** | | | | **In-/exclusion** | **Applicability criteria** | | | **Selection** |
| --- | --- | --- | --- | --- | --- | --- | --- | --- | --- |
| **Study included in ti/ab screening (study: 1^st^ author et al. year; Guideline: Organization – first 3 words of title)** | **Focuses on the airport / air travel setting** | **Regards infectious disease management** | **prescribes preparedness and response tasks** | **Has a generic (a-specific) approach regarding inf diseases** | **Inclusion = (4x Yes) OR (3x Yes + 1x Partly); all others = exclusion** | **Aimed at professionals at airport level** | **Covers unique topics regarding other studies** | **Covers the full cycle of preparedness and response** |  |
| AIRSAN – Contact tracing – collaboration | Yes | Yes | No | Yes | Exclusion | - | - | - | - |
| AIRSAN - Remote risk assessment | Yes | Yes | No | Yes | Exclusion | - | - | - | - |
| Boucard A. [Year: unknown] | Yes | Yes | No | No/partly | Exclusion | - | - | - | - |
| CAPSCA - Guidelines for States | Yes | Yes | Yes | Yes | Inclusion | No | No | Yes | 1 |
| Capsca - Health Summit 2012 | Yes | Yes | No | Yes | Exclusion | - | - | - | - |
| CAPSCA - Template For An Aviation | Yes | Yes | Yes | Yes | Inclusion | No | No | No | 0 |
| Cenciarelli O et al. 2014 | Yes | Yes | Partly | No | Exclusion | - | - | - | - |
| Chiu HH et al. 2014 | Yes | Yes | No | Yes | Exclusion | - | - | - | - |
| Dausey DJ et al. 2016 | Yes | Yes | No | Partly | Exclusion | - | - | - | - |
| ECDC RAGIDA | Yes | Yes | Partly | Yes | Inclusion | Yes | No | No | 1 |
| Findlater A & Bogoch I 2018 | Yes | No | No | Yes | Exclusion | - | - | - | - |
| Glynn RW & Boland M. 2016 | Yes | Yes | No | No | Exclusion | - | - | - | - |
| Ghassemi P et al. 2018 | Yes | Yes | No | Yes | Exclusion | - | - | - | - |
| Gold L et al. 2018 | Yes | Yes | No | Yes | Exclusion | - | - | - | - |
| Gosadi IM et al. 2015 | Yes | Yes | No | Partly | Exclusion | - | - | - | - |
| Harvey J et al. 2014 (abstract) | Yes | Yes | Unknown | Unknown | Exclusion | - | - | - | - |
| Huizer YL et al. 2014 | Yes | Yes | Partly | Yes | Inclusion | Yes | No | No | 1 |
| Kraus J et al. 2014 | Yes | Yes | Yes | Yes | Inclusion | Yes | No | No | 1 |
| Kraus J et al. 2016 | Yes | Yes | Yes | Yes | Inclusion | Yes | No | No | 1 |
| Marienau KJ et al. 2015 | Yes | Yes | No | Yes | Exclusion | - | - | - | - |
| Martin G & Boland M. 2018 | Yes | Yes | No | Partly | Exclusion | - | - | - | - |
| Memish ZA et al. 2014 | No | Yes | No | Yes | Exclusion | - | - | - | - |
| Nable JV et al. 2015 | Yes | Partly | No | No | Exclusion | - | - | - | - |
| Naouri D et al. 2016 | Yes | Partly | No | No | Exclusion | - | - | - | - |
| WHO 2012 – International Health | Yes | Yes | Yes | Yes | Included | Yes | Yes | Yes | 3 |
| WHO 2014 – Coordinated public | Yes | Yes | Partly | Yes | Included | Yes | Yes | No | 2 |
| WHO 2016 – Handbook for | Yes | Yes | Yes | Yes | Included | Yes | Yes | Yes | 3 |
| WHO 2019 - Action plan | No | Yes | Yes | No | Exclusion | - | - | - | - |
| Wilhelmi J. 2019 (unaccessable) | Unknown | Unknown | Unknown | Unknown | Exclusion | - | - | - | - |

- = not applicable.
