# Supplementary material for: Development of a competency profile for professionals involved in infectious disease preparedness and response in the air transport public health sector
Source: PLoS One. 2020 May 21;15(5):e0233360. doi: 10.1371/journal.pone.0233360 (PMC7241746; doi:10.1371/journal.pone.0233360)
Supplement: S2 File — (DOC) [file pone.0233360.s002.doc]

**S2. Full list of extracted competencies from literature**

**Competency domains**

**1. Policy development**

- 1. Public health policy
     1. Be familiar with the epidemiology of communicable diseases in order to guide public health policy
     2. Regularly assess and, as needed, clarify existing policies and/or recommend/advocate measures and communicate them to health officials at the ministry level, border control officials, and others.
     3. Before the response operation, ensure regular assessments of legal frameworks and propose/advocate measures to address gaps.
     4. The importance of alignment and interoperability of POE plans with the national public health and emergency response framework.
     5. Before the response operation, assess if the implementation of strategies, plans, and action plans requires any changes in these plans and strategies.
     6. Before the response operation, identify which triggers will require key decisions during outbreak response (keeping in mind that triggers may need to be modified to fit specific situations).
     7. Before response activities are taken, regularly review, test, and update the standard operating procedures and ensure that a multi-unit task force is available for the coordination and integration of relevant sectors during response operations.
     8. Review the evidence on current or impending outbreaks; propose and advocate adaptations to policies as needed.
     9. Communicate policy/guidelines, weigh benefits and costs, understand concerns about implementation, and adapt policies related to border control.
     10. Continuously evaluate evidence on threats; communicate if border control policies need to be adapted.
     11. Share information with response managers and health officials at the ministry level to support decisions about appropriate countermeasures.
  2. Document development
     1. Establishing and maintaining a Public health emergency contingency PHEC plan.
        1. Ensure alignment and interoperability between emergency response plans at the local, national and international levels.
        2. Update required national policy to support the PHECP
        3. Support the establishment and building of relationships between public health authorities and concerned POE stakeholders, agencies and service providers
        4. Development, testing and evaluation of a PHECP
        5. Procedures for monitoring the volume of movements of conveyances and travellers per conveyance through the PoE should be established.
     2. Standard operating procedures
        1. Periodically assess existing policies, plans and measures and communicate changes to relevant actors.
        2. Periodically assess the needs for changes in strategies and plans, and action plans, standard operating procedures (SOPs) for implementing them.
        3. Periodically review recommended triggers for key decisions during responses (keeping in mind that triggers may need to be modified to fit specific situations).
        4. Develop evidence-based guidelines for surveillance, prevention and control of communicable diseases and other public health events.
        5. Collect and analyse the relevant documentation for the development of an infection control procedure.
        6. Contribute to set a policy for the implementation and revision of infection control guidelines and recommendations according to the Standard Operating Procedures (SOPs);roles and responsibilities of supervisor, trainers, link professionals.
        7. Provide healthcare workers with clinical guidelines for emerging infections from abroad, especially those that may be carried by travellers and the severely contagious.
        8. Before the response operation, ensure the adequacy of plans for financing and credentialing of staff during

emergency situations.

- - - 1. Plan for the demobilisation and recovery of the healthcare workforce after a response operation.
      2. Continuously create and update an incident management plan that adapts existing policies to the situation at hand.
      3. Participate in the implementation of plans which ensure the continuity of operations.
      4. Before the response operation, identify key assumptions behind plans, identify untenable assumptions, and advocate changes as needed.
      5. Before the response operation, ensure adequate preparations for implementing health screening at borders;also ensure that response measures to a public health emergency can be taken right at the point of entry.
      6. Advocate the development of plans for joint task forces or other entities which can share information across disciplines.
    1. Interoperability of plans
       1. The interface between NaPHA or PHO’s and non-health related service providers, e.g. port, airport, ship and aircraft operators, which will have their own emergency plans is critical
  1. Communication
     1. Continuously inform public health emergency response managers about the threat so that the incident management plan can be updated.
     2. Communicate with political decision makers to mobilise needed resources, communicate current knowledge and uncertainties, and solicit guidance.
     3. Develop strategies to communicate with professionals who have different skills and knowledge levels; develop strategies to communicate with partner organisations to ensure a coordinated response.
  2. Roles and responsibilities
     1. Understand the roles and responsibilities of local, national and international organizations involved in infectious disease control.
     2. Assess the adequacy of mutual aid mechanisms and multi-disciplinary taskforces.
     3. Identify strategies to engage with government leaders in order to integrate government priorities and community interests; address concerns that surface during the emergency response.
     4. Play a facilitation role in the multisectoral collaboration that is required to build an effective PHECP.
  3. Laws, regulations and guidelines
     1. Periodically assess the legal frameworks and address gaps
     2. Identify strategies to engage with government leaders in integrating government priorities with community interests and concerns during the emergency response.
     3. Before the response operation, ensure that key partners are familiar with applicable laws, key roles,resources, information needs, and planning assumptions.

1. **Legal and ethical dimensions of international public health response**
   1. International
      1. Support the building of core capacities at designated POE
      2. The importance of supporting core capacity-building to ensure robust response capability required by designated POE
      3. Support the harmonization of regional and global emergency plans
      4. The specific requirements of IHR (2005) as they pertain to designated POE so that those aspects can be included in POE plans.
      5. To help NaPHA and PHOs better understand compliance and core capacity requirements under IHR (2005), WHO developed a Core Capacity Assessment Tool that can assist them in obtaining a baseline measurement of current capacity and gaps.
      6. Decision instrument (IHR annex 2) = to determine if an event may constitute a public health emergency of international concern
      7. The convention on International Civil Aviation issued by the International Civil Aviation Organization.
      8. Air traffix Management documents
   2. ICAO & CAPCA
      1. NaPHA should link to relevant international organizations and agencies (including WHO, the international Civil Aviation Organization (ICAO) and the international Maritime Organization (IMO), when needed.
      2. The PHECP should take into account mandatory requirements imposed by other relevant bodies, e.g. relevant ICAO standards and guidance.
      3. Implement the relevant ICAO standards and Recommended practices
   3. National legal authorities
      1. The PHECP should also take into account the context of a country in terms of government institutional arrangements and private sector involvement.
   4. Regional legal authorities
   5. Communication
   6. Roles and responsibilities
      1. NaPHA should be part of international networks of POE contacts
   7. Laws, regulations and guidelines
2. **Communication on public health events**
   1. Travellers, the public and media
      1. Public
         1. Demonstrate the capability to communicate to public, scientific community and decision makers.
         2. Apply principles of scientific communication to peers, stakeholders and media/public.
         3. Use most appropriate content and trusted channels of communication across population groups
         4. Identify data gathering mechanisms to understand and monitor the informational needs of the population.
         5. Prevent and counter misinformation.
         6. Proactively address the needs of the news media and the general public.
         7. Provide information to the public on the roles and responsibilities of the various organisations involved in the response operation; try to understand the public’s perception of the emergency.
         8. Identify communication mechanisms that are trusted by the public, partners, and community influencers.
         9. Empower the public to participate in open discussions; involve the public in decisions relevant to public health threats.
      2. Departing travelers
         1. The aviation sector provides information regarding medical clearance for travellers with health conditions that may impact their suitability for air travel.
         2. WHO and public health authorities provide comprehensive travel health information online for travellers to consider in planning a journey.
      3. In-transit and arriving travellers
         1. Travellers who are ill, have been exposed to a potential health risk during travel, or are arriving in an area where an illness or event has occurred may be asked to provide personal contact information in case future follow-up is required.
         2. They may also be provided with information on any potential risk. This includes travellers who are making connections between terminals or are transiting between different modes of transportation (from airline to rail or cruise ship)
      4. Emergency Risk Communication
         1. Understand and implement the principles of risk communication.
         2. Apply the basic principles of risk communication, adjusting the message when presenting results of an investigation to different audiences: media, public, professionals and policy makers.
         3. Proactively work with communication experts to address the needs of media and the people (including rumours and social media)
         4. Integrate the results of the risk-assessment process in the messages
         5. Anticipate questions from the public and develop appropriate answers
         6. Understand and implement the principles of risk communication
         7. Identify strategies to facilitate the release of information (i.e. review outgoing messages in a timely manner)
         8. While the aviation sector may facilitate the public health risk communications exchange, public health authorities are responsible for providing the information to be disseminated.
      5. Roles and responsibilities
         1. Develop a common understanding of roles, resources and planning with key partners.
      6. Laws, regulations and guidelines
         1. Understand laws and regulations related to ERC
3. **Surveillance**
4. Surveillance
   1. Understand the components of surveillance systems and how these work
   2. Related combined microbiological and epidemiological knowledge in outbreaks, surveillance, or unusual events.
   3. Be familiar with transmission dynamics and spatial spread of infectious diseases.
   4. Conduct timely and accurate disease reporting in accordance with WHO requirements and consistent coordination with FAO and OIE.
   5. It is crucial to have clear criteria for defining the types of events that must be communicated to the public health surveillance system in order to avoid overwhelming it with unnecessary volumes of information.
5. Ongoing monitoring
   1. Understand the importance to monitor and assess the results.
   2. Perform descriptive analysis of public health surveillance data
   3. Interpret results and trends form public health surveillance data analysis, including time series.
   4. Conduct an epidemiological study, including writing a study protocol, conducting data management, reporting and presenting the results and recommending evidence-based interventions to decision makers.
   5. Establish and maintain indicator and event-based surveillance system(s) to detect public health threats.
   6. Establish and maintain electronic real-time reporting systems.
   7. Interpret information from existing surveillance in order to characterize affected population groups, and to monitor disease trends and the impact of control strategies.
6. Early warning and response
   - - Indicator based surveillance
   1. Understand international disease reporting requirements.
   2. Understand the relevance and tools for early detection of public health threats.
   3. To early, detect public health risks and events of all origins, in order to ensure that they are rapidly investigated and controlled. The organized mechanisms to reach this objective is referred to as Early Warning and response (EWAR)
   4. Pertinent information for EWAR should be reported timely to the national health surveillance system (NHSS) and beyond, as appropriate.
   5. PoE should promptly receive all pertinent information generated elsewhere that may contribute to their public health surveillance objectives such as to prevent and/or manage the importation and exportation of health hazards.
   6. To enable the early detection of events for their timely verification and the application of control measures.
      - Event-based surveillance
   7. EBS is defined as the organized collection, monitoring, assessment and interpretation of mainly unstructured ad hoc information regarding events or public health risks, which may represent an acute risk to human health
7. Roles and responsibilities
   1. Collaborate with local public health officials and the healthcare delivery system, initiate active surveillance to identify additional cases during an epidemiologic investigation.
8. Communication
   1. Public health surveillance contact points should be identified at each PoE
9. Laws, regulations and guidelines
   1. Be familiar with laws on surveillance and reporting of communicable diseases at national, EU level and globally (International Health Regulations)
   2. Apply relevant laws to data collection, management, dissemination and use of information.
   3. Case-definitions must be standardizes country-wide
10. Event detection and event notification
    - 1. Incident recognition
         1. Use event-based and indicator-based surveillance systems to detect health threats.
         2. Know when case reports or clusters require further investigation, and how to initiate such investigations.
      2. Detection and assessment
         1. Understand and evaluate the implications of national or international public health alerts for own member state.
      3. Risk characterization
         1. Identify as rapidly as possible the (possibly novel) agent responsible for a disease outbreak and their epidemiological characteristics.
         2. Is the potential public health impact of the event serious (known morbidity, disability, mortality related with this type of event)
         3. Is this disease a specific target of a national or international control programme?
         4. Will the information collected lead to public health action (contact tracing, other specific control measures, international reporting).
      4. Event detection and notification/information sharing
         1. Pre-travel: detection at the point of origin
            1. If a traveller with a potentially communicable disease is identified at the point of origin, the traveller should be advised to delay travel until they have recovered. If the traveller has a notifiable communicable disease that may pose a health hazard to the public, the public health authorities should be alerted for case management and contact investigation.
         2. During the boarding process
            1. Travellers

Unusual or severe illness in departing travellers may be detected by port health or other authorities. In this event, passengers may be interviewed or subjected to a health assessment before being allowed to board.

Passengers agents for airlines and airports are given training to help identify travellers who appear to be unfit to fly, either at the counter, in the passenger lounge prior to boarding or at the time of boarding.

Passengers agents should seek medical advice before allowing the ill passenger to check In or to board the aircraft

If a traveller refuses to delay his/her travel, the airline may exercise their right to refuse boarding.

- - - - 1. Cargo

If cargo is suspected of being a potential public health hazard, it may be refused entry of required to undergo further testing or decontamination prior to receiving permission to enter the country and be transported.

- - - 1. During flight
         1. If a medical emergency occurs, the cabin crew may be able to seek advice from a ground-based medical service provider or the assistance of a medically trained passenger on board
         2. In serious cases, the pilot in command may consider the diversion in order for the unwell passenger to receive the required treatment
      2. Upon arrival and/or during transit to next airport
      3. Final destination
    1. Roles and responsibilities
       1. Who needs to be informed about events detected, the response measures implemented, and their results (chain of command) )
       2. Who is responsible at the national level for receiving the information from the local or intermediate level health authorities?
    2. **Coordination between PoE and the national health surveillance system**
       1. NaPHA should be part of the national command and control structure for public health emergency response
       2. NaPHA and local PHOs should link to national or local surveillance and response systems
       3. NaPHA should link to the NFP
       4. It is important to understand how the local POE public health authorities integrate with, report to and communicate with the national command and control structure
       5. It is essential for NaPHA to form a link with the national command and control structure to share vital information related to the POE and to provide health-related technical input and advice on the POE.
    3. Communication
       1. Communication with healthcare providers
          1. Before the response operation, establish rapid communication channels within national disease surveillance and healthcare professionals.
          2. For incident communication, draw on clinical personnel trained in risk communication or people involved in the incident, such as doctors or other clinicians.
          3. The competent authority responsible for cargo should work closely with the public health authorities to ensure appropriate methods for communication and cooperation are planned, tested and implemented at airports.
          4. Communication between the air crew and ground operations is necessary to ensure all parties are aware of the situation
       2. Reporting events tot the NHSS
          1. The information generated at PoE has to be reported on a timely basis to the national health surveillance system (NHSS)
       3. Information to travellers
    4. Laws, regulations and guidelines
       1. The pilot should notify air traffic control, as per ICAO provisions of any suspected cases of communicable diseases or evidence of a public health risk on board.

1. Risk profile of a country
   1. Governmental structure of the country regarding PoE
   2. Integrate and interpret information from a variety of local, national, and international sources regarding contaminants in air, soil and water.
   3. NaPHA and local PHOs should link to relevant port and airport authorities as well as air and maritime industry officials, as appropriate
   4. PHECP should take into account the risk profile of a country in terms of types and numbers of POE, quantity of international traffic, vectors and hosts present, and vulnerability of the area near the POE to vectors or disease
   5. PHECP should take into account resources that a country can provide and the resources available at each POE.
   6. Selection criteria for identifying events to be covered by surveillance should also consider the local context, including epidemiological patterns, vulnerability, available control measures and official priorities.
   7. Countries may have to identify other public health risks of interest to them at national level as well as to individual PoE.
2. **Human resources development**
   1. Training
      1. Training
         1. Periodically practice and test the ability to make decisions under uncertainty.
         2. Identify training needs, planning and organizing courses.
         3. Moderate case studies, give lectures and perform pedagogical teaching.
         4. Design/create a case study
         5. Before the response operation, practice and test the ability to make decisions under uncertainty..
         6. Develop protocols and test/exercise processes for health emergency operations and their activation.
         7. Provide training; include healthcare providers in drills and exercises to test communication lines and avoid communication problems.
         8. Before the response, train staff members in confidentiality policies, chains of evidence, and security issues relating to the exchange of information between partner organisations.
         9. Advocate regular multi-country exercises to improve the ability to communicate with partners.
         10. Develop a briefing schedule that includes all the required skills needed for specific roles for response personnel
         11. Training should be provided for conveyance operators, customs, and other personnel who have initial contact with travellers, to enable them to recognize key symptoms and signs of events among travellers.
      2. Exercises
         1. Implement lessons learned from planned exercises
         2. Advocate regular multi-discipline exercises to improve communication with staff and partners.
         3. Test the ongoing adequacy of the response plan
         4. Practice the public health operational response and identify the resources and roles required in a real-life public health emergency
         5. Test knowledge of legislation and powers
      3. Evaluation
         1. After each exercise or emergency event, conduct a formal review and update your plans accordingly with the key lessons learnt
   2. Harmonizing practices
      1. Understand the importance of multidisciplinary collaboration during epidemiological studies and outbreak investigations, including the one-health approach in zoonoses.
   3. Team resource management
      1. Before the response, establish trust with healthcare providers through feedback loops and ongoing two-way communication.
      2. Identify elements of stress management
      3. Be an effective team member, adopting the role needed to contribute constructively to the accomplishment of tasks by the group.
      4. Mutually identify those interests that are shared, opposed or different with the other party to achieve good collaboration and conflict management.
   4. Roles and responsibilities
   5. Communication
      1. Communicate effectively with persons from a multidisciplinary background, authorities, the public and the media in the form of publications, reports, interviews, and oral presentations.
   6. Laws, regulations and guidelines
3. **Health services**
   1. Preventive services
      1. Before an event, plan for the storage and stockpiling of vaccines and prepare for medical and non-medical countermeasures.
      2. Draw upon the work of surveillance networks to identify potential events that may indicate the need for the implementation of preventative services plans.
      3. Ensure that plans are in place for mass vaccinations and mass prophylactic medication distribution.
   2. Medical surge
      1. Prior to an event, work in tandem with clinicians to develop medical surge plans for various threats.
      2. Ensure that plans across the continuum of care have been communicated to the clinical staff to effectively manage surge needs.
      3. Plan for combining resources at national and local levels (e.g. cross-border sharing of clinicians if a hospital reaches capacity).
   3. Management of countermeasures, supplies and equipment
      1. Work with health personnel to identify the best medical countermeasures based on risk and threat; relay the results of these conversations
   4. Roles and responsibilities
      1. Identifying competent authorities and responsible agencies for each preventative measure.
      2. To inform competent authorities at PoE, and at all relevant levels in the health system and other sectors (e.g. customs, animal health, conveyance, operators), and to assist them in adopting preventive measures, investigations, management and follow up of events.
      3. What conveyances run at the PoE
   5. Laws, regulations and quidelines
      1. Identifying and updating national guidelines for **these** measures in line with relevant international guidelines.
      2. Coordinating with relevant stakeholders and agencies to ensure harmonized quality implementation and evaluation of these measures.
4. **Immediate arrangements for travelers and other stakeholders**
   1. Event verification and preliminary risk assessment
      1. In flight
         1. Infection control
         2. General considerations
      2. Upon arrival
   2. Personal Protective Equipment
      1. Identify appropriate decontamination strategies/personal protection and their applicability in field situations.
      2. Before a response operation, relay to healthcare workers the importance of their role in public health emergencies and support their personal preparedness and that of their families.
      3. Arrangements with relevant sectors t**o access to specially designated equipment and supplies and provision of trained personnel for special measures such as decontamination**, when required.
   3. Information sharing and possible activation of contingency plans
      1. Port health and first responders
      2. Immigrations and customs authorities
      3. Support services
   4. Port health assessment
   5. Transportation of travellers
      1. Relationship between PHOs and transport services providers, such as an ambulance service, which facilitates the rapid transport of suspected cases of an infectious disease (e.g. ill travellers to a local healthcare facility (e.g. a designated hospital)
   6. Diversion, parking, cleaning and disinfection of aircraft
      1. If evidence of a public health risk is founds on board a conveyance and the competent authority is not able to carry out the control measures required, the affected conveyance may nevertheless be allowed to depart, on condition that, at the time of departure, the competent authority informs its counterpart at the next known PoE of the evidence found and of the control measures required.
   7. Quality improvement
      1. Contribute to the integration of infection control activities within the healthcare organization’s quality promotion and patient safety programs
      2. Recognise security issues
   8. Roles and responsibilities
   9. Communication
      1. Communicate the necessity of measures to mitigate personal risks for the public health professionals.
   10. Laws, regulation and guidelines
5. **Risk assessment**
   1. Impact assessment
      1. Perform a risk assessment and continuously review as further information becomes available.
      2. Understand risk analysis frameworks, with the elements of risk assessment, risk management and risk communication.
      3. Understand the impact of control strategies
      4. Perform a risk assessment
      5. The rapid Risk Assessment of Acute Public Health Events is a useful reference on the risk assessment process.
   2. Roles and responsibilities
   3. Communication
      1. Communicate the results and implications of risk assessments to the relevant stakeholders
      2. Apply the results of international risk assessments to own member state.
      3. Communicate the results and implications of risk assessments for their own Member State to policy makers with different backgrounds.
      4. Communicate the results and implications of risk assessments to those responsible for emergency risk communication.
      5. To provide data to competent authorities for risk assessment of events.
   4. Laws, regulations and guidelines
      1. NaPHA should link to the national structure or mechanism responsible for public health risk assessment
   5. Diagnostics
      1. Recognise the use and limitation of diagnostic and typing methods and their interpretation in patient diagnosis, outbreak investigations, surveillance and epidemiological studies.
      2. Understand the principle of safe specimen sampling strategies for disease surveillance and for outbreak detection and control, both in humans and in animals.
      3. Interpret the diagnostic and epidemiological significance of reports from laboratory tests
      4. Conduct WHO core tests
      5. Have the biological, clinical, and epidemiological knowledge needed to characterize (potentially novel) pathogens and other agents responsible for an outbreak disease.
6. **Public health response**
   - 1. Ability to set up priorities based on medical evidence
     2. Identify public health priorities in complex emergency situations
   1. Outbreak investigation
      1. Be familiar with the steps of an outbreak
      2. Describe an outbreak in terms of person, place and time in order to generate hypothesis about its cause or risk factors
      3. Conduct outbreak investigations to identify pathogens and other agents, characterize affected population groups, and sources of exposure.
      4. Collaborate with local health officials, healthcare providers, and others to conduct outbreak investigations and epidemiological studies.
   2. Public health containment strategies
      1. Use evidence based methods to identify and recommend control and preventive measures to control an outbreak
   3. Travel advisories/health awareness campaigns
      1. Travel advisories
      2. Social mobilization (health awareness) campaigns
   4. Airport sanitation, vector control and disinsection
      1. Sanitation at airport
         1. Arrangements with relevant agencies (public or private**) to apply recommended measures for disinsection, derating, disinfection and decontamination**, when required.
      2. Sanitation on board aircraft
      3. Vector control
         1. Understand the importance of multidisciplinary collaboration during epidemiological studies and outbreak investigations, including the one-health approach in zoonoses.
         2. To prevent the international dissemination of vectors and reservoirs and the spread of vector-borne diseases.
      4. Disinsection
      5. Cargo and baggage
         1. Monitoring that baggage, cargo, containers, conveyances, goods, postal parcels and human remains carried through the PoE are free of sources of infection or contamination.
         2. Applying public health measures (e.g. inspections of conveyances, vector control, medical examination of travellers, disinfection, decontamination, insect control, and ratting)
         3. Conveyance operators shall facilitate the provision of relevant public health information requested by the state party,
   5. Border controls
      1. Entry and exit screening
         1. Arrangements with relevant agencies to **set up a system and procedures for quickly applying entry of exit controls (including screening) for arriving and departing travellers,** when required.
      2. Requirement for vaccination or other prophylaxis
      3. Use of screening technologies
      4. Syndromic surveillance
      5. Enhanced surveillance
         1. Develop and implement plans for border screening for known pathogens of international concern.
         2. Be able to use data products from epidemiologists in providing advice in the development of trade and travel restrictions as tools of population-based disease control.
      6. Public health declarations
         1. Competent authorities may require the health part of the Aircraft General Declaration, and from the pilot in command of an aircraft or the pilot’s agent, any information relating to health conditions on board during an international voyage.
      7. Public health risk and medical assessments
      8. Quarantine and isolation
         1. Port or airport authorities or relevant agencies to provide appropriate space for **interviewing suspected or affected person.**
         2. Relevant facilities (away from point of entry) to provide for the **assessment** and, if required, quarantine of suspect travellers.
   6. Contact tracing/investigation
      1. Be familiar with transmission dynamics and spatial spread of infectious diseases
      2. Establish reliable systems for disseminating case definitions to standardise both the diagnosis and the reporting of case numbers (e.g. confirmed, suspected, probable, or possible cases).
   7. Significant interference with international traffic
      1. Operational relationship between PHOs and the airport operator and/or air traffic services provider to determine the appropriate parking stand for an incoming affected aircraft.
   8. Health measures for events related to risk in the environment
   9. Response measures to events of unknown etiology including chemical and radiological hazards
      1. To prevent or manage the importation and exportation of health hazards (including diseases and their agents) in a country.
   10. Public health measures for animals
   11. (Use of specific health measures to ensure the safe handling an transport of human remains)
       1. Monitoring that baggage, cargo, containers, conveyances, goods, postal parcels and human remains carried through the PoE are free of sources of infection or contamination.
   12. Roles and responsibilities
       1. Share relevant information with healthcare, infection control, and patient transport experts, and solicit their feedback.
       2. Identify interdisciplinary needs between health-care professionals and front-line responders.
       3. Identify key partners and develop a common understanding of roles, resources, planning assumptions,risks/vulnerabilities, and information that should be shared during response operations.
       4. Recognize the important role of NaPHA and PHOs, prior to and during an response
       5. Recognize the requirement of a direct operational link between NaPHA and the NFP within the National Public Health authority, such as the Ministry of Health, in coordinating the national public health emergency response.
       6. Operational relationships are needed among the NaPHA and emergency or incident command and response teams
       7. Operational relationships are needed among the emergency operations centre for POE response and POE medical, security, customs, immigration and quarantine officials
       8. Operational relationships are needed among the emergency operations centre for POE response and the local and regional medical facilities
       9. Operational relationship are needed among the emergency operation center for POE response and suppliers of medical, psychosocial and logistical services
       10. Operational relationships are needed among emergency operations centre for POE response and providers of transport to hospitals and quarantine facilities
       11. Operational relationships are needed among the emergency operations center for POE response and other local and national emergency operations and command structures.
       12. During a PHEIC, NaPHA or PHOs should become part of the national or local command and control structure by attaching as a direct report to the incident commander
   13. Monitoring and evaluation of event response
   14. Communication
       1. Recognize the importance of ensuring participation in effective international communication including sharing information during PHEIC.
       2. During the response, anticipate resource needs on an ongoing basis and communicate them to relevant decisions makers.
       3. During the response, communicate with political decision makers to mobilize needed resources, communicate current knowledge and uncertainties, and solicit guidance.
       4. Coordinate response using communication mechanisms and other tools.
       5. During the response operation, anticipate resource needs and communicate them to relevant decision makers.
   15. Laws, regulations and guidelines
       1. Understand national, European and World Health Organization (WHO) rules and regulations regarding biosafety and biosecurity and understand how these may influence field situations.
       2. Apply relevant laws to data collection, management, dissemination and use of information.
       3. Adhere to ethical principles regarding data protection and confidentiality regarding any information obtained as part of professional activity.
       4. Documentation of arrangements made with relevant agencies for response measures, such as patient treatment, isolation and quarantine
       5. Pilots in command of aircraft, or their agents, shall make known to the airport control as early as possible any cases of illness indicative of a disease of an infectious nature or evidence of a public health risk on board. This information must be immediately relayed to the competent authority or airport. In urgent situations, such information should be communicated directly by the officers or pilots to the relevant port of airport authority.
       6. Criteria for implementing contact tracing; the completion of a public health passenger locator form by passengers and crew members who have been in contact with a case AND
       7. Passenger Name List – PNL (‘Passenger Manifest’): in case of an event aboard an aircraft, States Parties can require the aircraft conveyer to present the PNL. Which should provide the names of all passengers aboard the aircraft.
   16. (Safe transport of infectious substances)
       1. Operational relationships are needed among the emergency operations center and cargo/freight handlers and hazardous material crews
7. **Evaluation (lesson learned)**
   1. Roles and responsibilities
   2. Communication
   3. Laws, regulations and guidelines
8. **Technical competencies**
   1. Advising and guiding counterparts in the national command and control structure
   2. Roles and responsibilities
      1. Support the enhancement of technical competencies of NaPHA and local PHOs
      2. The promotion of technical upskilling of POE health officials to enable them to provide better advice and guidance during an emergency.
      3. For information sharing to be successful, it may be necessary to upskill and increase the technical competencies of NaPHA and local PHOs to allow them to perform in this capacity.
   3. Communication
   4. Laws, regulations and guidelines
9. **Ethics and integrity**
   1. Airport culture competency
      1. Address cultural and societal barriers in the cognitive processing and compliance with recommended behaviors.
      2. Identify strategies to overcome linguistic barriers, e.g. request local assistance
   2. Roles and responsibilities
   3. Communication
   4. Laws, regulations and guidelines
      1. Adhere to ethical principles regarding human welfare when planning studies, conducting research, and collecting, disseminating and analyzing data.
      2. States parties are obliged to collect and handle health information containing personal identifiers in a confidential manner. However, states parties may disclosure and process personal data when it is essential for the purposes of assessing and maintaining a public health risk, subject to particular conditions.
