# Supplementary material for: Development of a competency profile for professionals involved in infectious disease preparedness and response in the air transport public health sector
Source: PLoS One. 2020 May 21;15(5):e0233360. doi: 10.1371/journal.pone.0233360 (PMC7241746; doi:10.1371/journal.pone.0233360)
Supplement: S3 File — (DOC) [file pone.0233360.s003.doc]

**S3. Reduced list of extracted competencies from literature**

**Communication, collaboration and professionalism during the entire process.**

| **CanMEDS rolls** | **Competencies** |
| --- | --- |
| **Communication** | - Apply principles of scientific (risk) communication (appropriate content and trusted channels) to peers, stakeholders and media / public. - Establish trust with healthcare providers through rapid communication channels and ongoing two-way communication. - Recognize who needs to be informed about events detected on board, the response measures implemented, and their results (chain of command) - Continuously evaluate evidence on threats: communicate if border control policies need to be adapted. |

| **CanMEDS rolls** | **Competencies** |
| --- | --- |
| **Collaboration** | - Be an effective team member, adopting the role needed to contribute constructively to the accomplishment of tasks by the group. - Mutually identify those interests that are shared, opposed or different with the other party to achieve good collaboration and conflict management. - Understand the importance of multidisciplinary collaboration during epidemiological studies and outbreak investigations, including the one-health approach in zoonoses. - Participate in the implementation of plans, which ensure the continuity of operations. - Collect and integrate the facts of an event, based on information from multiple sources including the traveller, the aircraft operator, ground-based medical services for aircraft in flight (when available) or the agent responsible for the baggage or cargo. |

| **CanMEDS rolls** | **Competencies** |
| --- | --- |
| **Professionalism** | - Adhere to ethical principles regarding data protection and confidentiality regarding any information obtained as part of professional activity. - Decide when disclosure and process of personal data is essential for the purposes of assessing and maintaining a public health risk. - Treat crewmembers and passengers with respect for their dignity, human rights, and fundamental freedoms and minimize any discomfort or distress associated with measures such as quarantine and isolation. |

**General competencies**

| **CanMEDS rolls** | **Competencies** |
| --- | --- |
| **Medical / Public health- specific knowledge and skills** | - Recognize a potentially communicable disease by key symptoms and signs of events among travellers. - Advise travellers with a potentially communicable disease about the continuation of their journey. - Execute vaccination of other prophylaxis after the agreement of the traveller or his/her parents of guardians. - Be familiar with laws on surveillance and reporting of communicable diseases at national, EU level and globally (International Health Regulations). - Be familiar with standards and recommended practices concerning infectious disease control from national and international aviation organizations (IATA, ICAO and CAPSCA) |
| **Organization / Policy development / Roles and responsibilities** | - Develop evidence-based guidelines for surveillance, prevention and control of communicable diseases and other public health events. - Before the response operation, assess if the implementation of strategies, standard operating procedures (SOPs) and action plans requires any changes. - Understand the roles and responsibilities of local, national and international organizations involved in infectious disease control. |
| **Science / epidemiology** | - Be familiar with transmission dynamics and spatial spread of infectious diseases. - Integrate and interpret information from a variety of local, national, and international sources regarding contaminants in air, soil and water. |

**PREPAREDNESS**

**Training – This section is aimed at professionals who conduct trainings**

| **CanMEDS rolls** | **Competencies** |
| --- | --- |
| **Medical / Public health – specific knowledge and skills** | - Design/create a case study - Moderate case studies, give lectures and perform pedagogical teaching. - Provide training; include healthcare providers in drills and exercises to test communication lines and avoid communication problems. |
| **Organization / Policy development / Roles and responsibilities** | - Identify training needs, planning and organizing courses. - Implement lessons learned from planned exercises - Periodically practice and test the ability to make decisions under uncertainty. |
| **Science / epidemiology** |  |

**Surveillance**

| **CanMEDS rolls** | **Competencies** |
| --- | --- |
| Medical / Public health specific knowledge and skills | - Use event-based and indicator-based surveillance systems to detect health threats. - Understand the relevance and tools for early detection of public health threats. - Understand the components of surveillance systems and how these work |
| Organisation / Policy development / Roles and responsibilities | - Evaluate pertinent information generated by all involved parties that may contribute to public health surveillance objectives - Know when case reports or clusters require further investigation, and how to initiate such investigations. |
| Science / epidemiology | - Perform descriptive analysis of public health surveillance data - Interpret information from existing surveillance in order to characterize affected population groups, and to monitor disease trends and the impact of control strategies. - Conduct an epidemiological study, including writing a study protocol, conducting data management, reporting and presenting the results and recommending evidence-based interventions to decision makers. |

**Contingency planning**

| **CanMEDS rolls** | **Competencies** |
| --- | --- |
| Medical / Public health specific knowledge and skills | - Before the response operation, identify which triggers will require key decisions during outbreak response (keeping in mind that triggers may need to be modified to fit specific situations). |
| Organization / Policy development / Roles and responsibilities | - Support the building of core capacities at designated POE, and understand the importance of supporting core capacity-building - Develop, test and evaluate a Public Health Emergency Contingency Plan (PHECP) - Before an event, plan for the storage and stockpiling of vaccines and prepare for medical and non-medical countermeasures. |
| Science / epidemiology | - Provide healthcare workers with clinical guidelines for emerging infections from abroad, especially those that may be carried by travellers and the severely contagious. |

Risk assessment

| **CanMEDS rolls** | **Competencies** |
| --- | --- |
| **Public health / medical specific knowledge and skills** | - Determine when a risk assessment should be carried out, and appropriate measures should be taken. - Perform a risk assessment - Understand risk analysis frameworks, with the elements of risk assessment, risk management and risk communication. - Continuously review the risk assessment as further information becomes available. |
| **Organisation / Policy development / Roles and responsibilities** | - Understand the impact of control strategies |
| **Science / epidemiology** | - Interpret the diagnostic and epidemiological significance of reports from laboratory tests - Understand the principle of safe specimen sampling strategies for disease surveillance and for outbreak detection and control, both in humans and in animals. - Integrate and interpret information from a variety of local, national, and international sources regarding contaminants in air, soil and water. |

**RESPONS**

**Outbreak investigation**

| **CanMEDS rolls** | **Competencies** |
| --- | --- |
| Medical / Public health specific knowledge and skills | - Be familiar with the steps of an outbreak - Describe an outbreak in terms of person, place and time in order to generate hypothesis about its cause or risk factors - Conduct outbreak investigations to identify pathogens and other agents, characterize affected population groups, and sources of exposure. |
| Organisation / Policy development / Roles and responsibilities | - Identify who is responsible at the national level for receiving the information on the investigation from the local or intermediate level health authority. |
| Science / epidemiology | - Have the biological, clinical, and epidemiological knowledge needed to characterize (potentially novel) pathogens and other agents responsible for an outbreak disease. |

**Activation of the PHEC Plan**

| **CanMEDS rolls** | **Competencies** |
| --- | --- |
| Medical / Public health – specific knowledge and skills | - Identify public health priorities in complex emergency situations |
| Organisation / Policy development / Roles and responsibilities | - Identify key partners and develop a common understanding of roles, resources, planning assumptions, risks/vulnerabilities, and information that should be shared during response operations. - During the response, anticipate resource needs on an ongoing basis and communicate them to relevant decisions makers. - Prepare immigration / border personnel with information to protect themselves and healthy travellers, as required. - Prepare cleaning personnel (including aircraft cleaners) and ground handling service with information regarding the public health event to protect themselves and healthy travellers as required. - Prepare cargo and baggage handlers with information about the public health event, including identification of any potentially affected baggage or cargo to protect themselves and healthy travellers as required. |
| Science / epidemiology | - Draw upon the work of surveillance networks to identify potential events that may indicate the need for the implementation of preventative services plans. |

Public health measures

| **CanMEDS rolls** | **Competencies** |
| --- | --- |
| **Medical / Public health specific knowledge and skills** | - **Identify travellers who may be symptomatic with, or who were possibly exposed to a case of the illness of concern.** - **Determine triggers for appropriate public health measures, such as travel restrictions, quarantine, treatment and isolation, that are commensurate with the risk and do not unduly interfere with international travel.**    - Analyse the costs of the interventions and resulting liabilities   - Arrange the use of quarantine by scientific evidence and expert public health opinions to avoid contradictory or unnecessary restrictions of individuals.   - Decide when to quarantine travelers suspected of being ill for a period to ensure that there is no risk of spreading infection or contamination. - **Arrange the welfare of animals in transit and prevent the transmission of diseases among animal and between animal and humans.**   - Arrange logistic support for implementation of isolation and quarantine as well as access to health services for animals |
| **Organisation / Policy development / Roles and responsibilities** | - **Arrange medical advice before allowing the ill passenger to check in or to board the aircraft.** - **Asses the conditions for refusing travellers to board an aircraft.** |
| **Science / epidemiology** | - **Use evidence based methods to identify and recommend control and preventive measures to control an outbreak**    - Arrange specialist advice to determine the necessary control measures and evaluate their effectiveness - **Be able to use data products from epidemiologists in providing advice in the development of trade and travel restrictions as tools of population-based disease control.** |

**Management of ill / exposed travellers**

| **CanMEDS rolls** | **Competencies** |
| --- | --- |
| Medical / Public health specific knowledge and skills | - Determine when personal protective equipment (PPE) is required. - Recognize guidance including the use of appropriate PPE to reduce the risk to themselves and other travellers.   - Identify appropriate decontamination strategies/personal protection and their applicability in field situations. - Arrange medical advice from a ground-based medical service provider or the assistance of a medically trained passenger on board.   - Designate one cabin crewmember to look after the ill traveller   - Handle all waste in accordance with regulatory requirements of guidelines to limit contamination - Conduct a public health observation of travellers who have been exposed or possible exposed in order to monitor his/her health status and generate personal contact information in case future follow-up is required.   - Assess health status of travellers from an affected region or who have been exposed to a potential public health risk during air travel.   - Perform a medical examination following the rules of IHR and national legislation for safety standards. |
| Organisation / Policy development / Roles and responsibilities | - Determine the appropriate parking stand for an incoming affected aircraft. - Determine the order of disembarkation of passengers - Arrange efficient port health staff in order to reduce the time that travellers spend on a board-affected aircraft. - Arrange a possible transfer to medical facility by ambulance and facilitate the rapid transport of suspected cases of an infectious disease - Identify space requirements for interviews and health assessments of arriving travellers. - Separate travellers from the concerned conveyance from other travellers during border control screening and asses if other relevant stakeholders are aware of the situation. |
| Science / epidemiology |  |

Contact tracing

| **CanMEDS rolls** | **Competencies** |
| --- | --- |
| Medical / Public health specific knowledge and skills | - Use reliable systems for disseminating case definitions to standardize both the diagnosis and the reporting of case numbers (e.g. confirmed, suspected, probable, or possible cases). - Systematically generate required information about the number of travellers such as those targeted for screening, screened, referred to secondary screening, and identified as confirmed cases - Determine when to request a Passenger Locator Form (PLF), from whom and where and how the information from these forms will be used and subsequently stored or destroyed. |
| Organisation / Policy development / Roles and responsibilities |  |
| Science / epidemiology |  |

**Handling goods, luggage, human-remains**

| **CanMEDS rolls** | **Competencies** |
| --- | --- |
| Medical / Public health specific knowledge and skills | - Recognize sources of infection in baggage, cargo, containers, conveyance, goods, postal parcels and human remains carried through the PoE - Recognize when to implement special handling of baggage or cargo from affected regions, including inspection, fumigation, other decontamination or possibly destruction   - Apply health measures to baggage, cargo, containers, conveyances, goods, postal parcels or humans remains in accordance with IHR (article 22) and ensure the effectiveness of these control measures.   - Determine when to adopt different procedures or use additional PPE when handling baggage from affected aircraft.   - Decide, based on ‘chain of custody’ documentation, whether suspect or contaminated cargo should be subjected tovisual X-ray or other screening and/or inspection.   - Decide upon further testing or decontamination of refused cargo is warranted. |
| Organisation / Policy development / Roles and responsibilities | - Identify specific requirements of the destination country for carrying human remains.   - Arrange required official documentation regarding the traveler’s identity and cause of death to accompany the coffin. - Conduct appropriate packaging and transport of possible suspected specimens of human and animal origin to protect from the risk of infection |
| Science / epidemiology |  |

Airport/aircraft sanitation

| **CanMEDS rolls** | **Competencies** |
| --- | --- |
| Medical / Public health specific knowledge and skills | - Determine, based on results of the inspection, if further public health measures are warranted such as disinfection, decontamination, disinsection or derating.   - Recognize when enhanced cleaning and disinfection or decontamination is warranted . - Determine if a public health risk exists based on examination of all aircraft facilities. |
| Organisation / Policy development / Roles and responsibilities | - Arrange satisfactory cleaning and disinfection by ground handling staff prior to the aircraft’s next flight. - Contribute to the integration of infection control activities within the healthcare organization’s quality promotion and patient safety programs |
| Science / epidemiology | - Prevent the international dissemination of vectors and reservoirs and the spread of vector-borne diseases |

**Recovery**

**Evaluation and recovery**

| **CanMEDS rolls** | **Competencies** |
| --- | --- |
| Medical / Public health specific knowledge and skills | - Clearly define goals and objectives of the evaluation. - Develop a formal evaluation of the response and share with all stakeholders, when the public health is under control of concluded. |
| Organisation / Policy development / Roles and responsibilities | - Deactivate the plan and return to recovery once the situation is under control or able to de de-escalated. - Plan for the demobilization and recovery of healthcare workers after a response operation - Update plans according the key lessons learnt after a formal review |
| Science / epidemiology |  |
