# Supplementary material for: Development of a competency profile for professionals involved in infectious disease preparedness and response in the air transport public health sector
Source: PLoS One. 2020 May 21;15(5):e0233360. doi: 10.1371/journal.pone.0233360 (PMC7241746; doi:10.1371/journal.pone.0233360)
Supplement: S1 Questionnaire — (PDF) [file pone.0233360.s005.pdf]

S5. Questionnaire National Data Collection

Rijksinstituut voor Volksgezondheid
en Milieu
Ministerie van Volksgezondheid,
Welzijn en Sport

Voortgangs balk

Competentieprofiel infectieziekte voorbereiding en bestrijding op luchthavens.

Introductie

U hebt in april een uitnodiging ontvangen om deel te nemen aan een onderzoek. Dit onderzoek wordt uitgevoerd door het **Rijksinstituut voor Volksgezondheid en Milieu (RIVM).** Het doel van dit onderzoek is het opstellen van een competentieprofiel voor professionals betrokken bij infectieziekte voorbereiding en bestrijding op luchthavens. Deze studie maakt deel uit van een promotie-onderzoek naar het versterken van preparatie- en responsecapaciteit van de zogenaamde points of entry (PoE) m.b.t. cross-border incidenten, in het kader van de **Healthy Gateways Joint Action**.

Achtergrond
De betekenis van een competentie is het vermogen of de kennis om een handeling bekwaam uit te voeren of een probleem juist op te lossen. Competenties zijn o.a. nodig voor het opstellen en evalueren van training programma's. Hoewel er algemene competentie profielen bestaan, ontbreekt een profiel specifiek voor infectieziekte voorbereiding en bestrijding op **luchthavens**. De afgelopen weken hebben we op basis van literatuur een eerste voorstel voor een competentielijst opgesteld.

Waarom krijgt u deze vragenlijst?
Wij hebben input uit de praktijk nodig om een zinvolle lijst te kunnen maken. Wij vragen u als professional daarom deze potentiele competenties te beoordelen.Het is belangrijk dat u daarbij in gedachten houd dat de competenties specifiek voor luchthavens moeten gelden. Dat doet u door:

- De competenties te beoordelen op relevantie voor infectieziektevoorbereiding en –bestrijding op luchthavens.
- De competenties eventueel te herformuleren. U kunt hierbij gebruik maken van het formuleer advies in de bijlage. Wij raden u aan dit uit te printen en naast u te houden tijdens het invullen.

Opbouw competentielijst
De vragenlijst bestaat uit drie processen: Voorbereiding (**Preparedness**), Reactie (**Response**) en Herstel (**Recovery**) met bijbehorende taken .Specifieke taken bestaan vervolgens uit 3 gebieden namelijk; **Medische / Publieke gezondheid kennis & vaardigheden**, **Organisatie en Wetenschap**.

Competenties op het gebied van **Communicatie, Samenwerking** en **Professionaliteit** gelden voor alle processen.

Het invullen van de lijst duurt ongeveer 20 tot 30 minuten.

Vervolg onderzoek: groepsdiscussie
Het onderzoek bestaat naast de vragenlijst ook uit een groepsdiscussie (consensus procedure) over de competenties die onder de deelnemers verschillend zijn gescoord.De discussie vindt plaats op donderdag 20 juni. Hier ontvangt u nog aanvullende informatie over. Voor die tijd ontvangt u tevens zowel uw eigen resultaten als de gemiddelde resultaten van alle deelnemers, in de vorm van een feedback rapport.

Hebt u vragen over de vragenlijst of het onderzoek?
Neem dan contact op met de onderzoeker [redacted]

* = Invoer verplicht

Verder >> **Tussentijds bewaren**

Rijksinstituut voor Volksgezondheid en Milieu
Ministerie van Volksgezondheid, Welzijn en Sport

Voortgangs balk

Informed consent

Uw deelname is vrijwillig en uw gegevens worden anoniem verwerkt. Meedoen is vrijwillig. Ook als u begint met meedoen, kunt u altijd nog stoppen.Neem hiervoor contact op met Rebekka Rebel via [redacted]

Met het aanklikken van onderstaand icoon gaat u akkoord met het verzamelen en gebruiken van enkele persoonsgegevens.
Het gaat om gegevens zoals uw geslacht, functie, en het aantal jaren werkzaam in uw huidige functie.
Gegevens zullen zorgvuldig en vertrouwelijk worden behandeld en zullen alleen voor deze studie worden gebruikt.
We zullen resultaten alleen in algemene termen rapporteren zonder een persoon te benoemen.
Deze informatie wordt 15 jaar bewaard, maar alleen de betrokken wetenschappers van het RIVM kunnen deze zien.
Lees voor het akkoord geven hier meer over in de privacyverklaring van het RIVM: https://www.rivm.nl/privacy
*

○ Ja, ik ga akkoord

* = Invoer verplicht

<< Terug | Verder >> | Tussentijds bewaren

Rijksinstituut voor Volksgezondheid en Milieu
Ministerie van Volksgezondheid, Welzijn en Sport

Voortgangs balk

Het formuleren van competenties

Voor u met de vragenlijst start geven wij u een korte instructie over de opbouw van competenties.
Competenties omvatten ten minste twee onderdelen;

1. Een werkwoord om het cognitieve niveau te specificeren.
2. Een onderwerp waarop de kennis, vaardigheid of houding betrekking heeft.

Aanbevolen:
3. Een bijwoord om de kwaliteit te bepalen

Voorbeelden

- Recognize a potentially infectious disease by key symptoms and signs of events among travellers.
- Rapidly facilitate the transport of suspected cases of an infectious disease.

Indien u herformulering nodig acht, maak dan gebruik van het formuleer advies in de bijlage.
Wij raden u aan dit uit te printen en naast u te houden tijdens het invullen.

* = Invoer verplicht

<< Terug | Verder >> | Tussentijds bewaren

Rijksinstituut voor Volksgezondheid en Milieu
Ministerie van Volksgezondheid, Welzijn en Sport

Voortgangs balk

Op de volgende pagina start de vragenlijst. Probeer de vragenlijst zo volledig mogelijk in te vullen.
We kunnen u ook telefonisch helpen [redacted]

We streven ernaar de vragenlijsten uiterlijk 3 juni te ontvangen.

* = Invoer verplicht

<< Terug | Verder >> | Tussentijds bewaren

Rijksinstituut voor Volksgezondheid en Milieu
Ministerie van Volksgezondheid, Welzijn en Sport

Voortgangs balk

Demografische vragen

Ik ben een

- ○ Man
- ○ Vrouw

Wat is uw huidige functie ten behoeve van infectieziekte voorbereiding en bestrijding op Schiphol?

Hoeveel jaar bent u werkzaam in uw huidige functie?

Met welke partijen werkt u ten behoeve vavn infectiziekte voorbereiding en bestrijding het meeste samen?

- ☐ GGD artsen
- ☐ GGD verpleegkundigen
- ☐ Airport Medical Services
- ☐ Vliegtuigmaatschappijen

Anders, namelijk;

In hoeverre voelt u zich ervaren met infectieziekte voorbereiding en response op luchthavens?

- ○ 1 (Zeer onervaren)
- ○ 2
- ○ 3
- ○ 4
- ○ 5 (Zeer ervaren)

Geef hieronder het email-adres waarmee u bent benaderd.

Indien u het persoonlijk feedback rapport met een ander e-mailadres wilt ontvangen, vermeld deze dan hieronder.

* = Invoer verplicht

<< Terug | Verder >> | Tussentijds bewaren

Rijksinstituut voor Volksgezondheid en Milieu
Ministerie van Volksgezondheid, Welzijn en Sport

Voortgangs balk

Vervolg vragenlijst

U heeft zonet demografische vragen beantwoord. De vragenlijst gaat nu verder met algemene competenties op het gebied van **Communicatie**, **Samenwerking** & **Professionaliteit.**

* = Invoer verplicht

<< Terug | Verder >> | Tussentijds bewaren

Rijksinstituut voor Volksgezondheid
en Milieu
*Ministerie van Volksgezondheid,
Welzijn en Sport*

Voortgangs balk

Communication

To what extent do you consider this competency as a relevant element for infectious disease preparedness and response at airports? On a 9-point Likert scale (1=totally irrelevant, 9 =totally relevant)

Apply principles of scientific communication and risk communication (appropriate content and trusted channels) to peers, stakeholders and media / public.

	1	2	3	4	5	6	7	8	9
*	○	○	○	○	○	○	○	○	○

If needed, please reformulate this competency

Establish trust with healthcare providers through rapid communication channels and ongoing two-way communication.

	1	2	3	4	5	6	7	8	9
*	○	○	○	○	○	○	○	○	○

If needed, please reformulate this competency

Zijn er op het gebied van communicatie (communication) nog competenties die u hier mist? Denk bijvoorbeeld aan specifieke kennis, vaardigheden of houding die bij communicatie belangrijk zijn? Schrijf hieronder uw suggesties op.

* = Invoer verplicht

<< Terug | Verder >> | Tussentijds bewaren

Voortgangs balk

Collaboration

To what extent do you consider this competency as a relevant element for infectious disease preparedness and response at airports? On a 9-point Likert scale (1=totally irrelevant, 9 =totally relevant)

Understand the importance of multidisciplinary collaboration during epidemiological studies and outbreak investigations, including the one-health approach in zoonoses.

	1	2	3	4	5	6	7	8	9
*	○	○	○	○	○	○	○	○	○

If needed, please reformulate this competency

Be an effective team member, adopting the role needed to contribute constructively to the accomplishment of tasks by the group.

	1	2	3	4	5	6	7	8	9
*	○	○	○	○	○	○	○	○	○

If needed, please reformulate this competency

Participate in the implementation of plans, which ensure continuity of operations.

	1	2	3	4	5	6	7	8	9
*	○	○	○	○	○	○	○	○	○

If needed, please reformulate this competency

Zijn er op het gebied van samenwerking (collaboration) nog competenties die u hier mist? Denk bijvoorbeeld aan specifieke kennis, vaardigheden of houding die bij samenwerking belangrijk zijn? Schrijf hieronder u suggesties op.

* = Invoer verplicht

<< Terug | Verder >> | Tussentijds bewaren

Rijksinstituut voor Volksgezondheid en Milieu
Ministerie van Volksgezondheid, Welzijn en Sport

Voortgangs balk

Professionalism

Adhere to ethical principles regarding data protection and confidentiality regarding any information obtained as part of professional activity.

	1	2	3	4	5	6	7	8	9
*	○	○	○	○	○	○	○	○	○

If needed, please reformulate this competency

Decide when disclosure and process of personal data is essential for the purposes of assessing an maintaining a public health risk.

	1	2	3	4	5	6	7	8	9
*	○	○	○	○	○	○	○	○	○

If needed, please reformulate this competency

Treat crewmembers and passengers with respect for their dignity, human rights, and fundamental freedoms and minimize any discomfort or distress associated with public health measures.

	1	2	3	4	5	6	7	8	9
*	○	○	○	○	○	○	○	○	○

If needed, please reformulate this competency

Zijn er op het gebied van professionaliteit (professionalism) nog competenties die u hier mist? Denk bijvoorbeeld aan specifieke kennis, vaardigheden of houding die bij professionaliteit belangrijk zijn? Schrijf hieronder uw suggesties op.

* = Invoer verplicht

<< Terug | Verder >> | Tussentijds bewaren

Rijksinstituut voor Volksgezondheid en Milieu
Ministerie van Volksgezondheid, Welzijn en Sport

Voortgangs balk

Preparedness

U heeft de algemene competenties op het gebied van **Communicatie**, **Samenwerking** & **Professionaliteit** zojuist beoordeeld.

Nu gaat de vragenlijst verder met taken die specifiek toebehoren onder **Preparedness**.
Dat zijn; **Training**, **Contingency planning** , **Surveillance**, **Risk assessment** en **Outbreak investigation.**

* = Invoer verplicht

<< Terug | Verder >> | Tussentijds bewaren

Rijksinstituut voor Volksgezondheid en Milieu
Ministerie van Volksgezondheid, Welzijn en Sport

Voortgangs balk

Training

To what extent do you consider this competency as a relevant element for infectious disease preparedness at airports? On a 9-point Likert scale (1=totally irrelevant, 9 =totally relevant)

Medical / Public Health - specific knowledge and skills

Moderate case studies, give lectures and perform pedagogical teaching.

	1	2	3	4	5	6	7	8	9
*	○	○	○	○	○	○	○	○	○

If needed, please reformulate this competency

Provide training, include healthcare workers in drills and exercises to test communication lines and avoid communication problems.

	1	2	3	4	5	6	7	8	9
*	○	○	○	○	○	○	○	○	○

If needed, please reformulate this competency

Organization / Policy development / Roles and Responsibilities

Identify training needs, planning and organizing courses.

	1	2	3	4	5	6	7	8	9
*	○	○	○	○	○	○	○	○	○

If needed, please reformulate this competency

Periodically practice and test the ability to make decisions under uncertainty

	1	2	3	4	5	6	7	8	9
*	○	○	○	○	○	○	○	○	○

If needed, please reformulate this competency

Zijn er op het gebied van training nog competenties die u hier mist? Denk bijvoorbeeld aan specifieke kennis, vaardigheden of houding die bij training belangrijk zijn? Schrijf hieronder uw suggesties op.

* = Invoer verplicht

<< Terug | Verder >> | Tussentijds bewaren

Rijksinstituut voor Volksgezondheid
en Milieu
Ministerie van Volksgezondheid, Welzijn en Sport

Voortgangs balk

Contingency planning

To what extent do you consider this competency as a relevant element for infectious disease preparedness at airports? On a 9-point Likert scale (1= totally relevant, 9=totally relevant)

Medical / Public Health specific knowledge and skills

Be familiar with standards and recommended practices concerning infectious disease control from national and international aviation organizations (IATA, ICAO and CAPSCA)

	1	2	3	4	5	6	7	8	9
*	○	○	○	○	○	○	○	○	○

If needed, please reformulate this competency

Before the response operation, assess if the implementation of strategies, standard operating procedures (SOPs) and action plans requires any changes.

	1	2	3	4	5	6	7	8	9
*	○	○	○	○	○	○	○	○	○

If needed, please reformulate this competency

Before the response operation, identify which triggers will require key decisions during outbreak response (keeping in mind that triggers may need modification to fit specific situations.

	1	2	3	4	5	6	7	8	9
*	○	○	○	○	○	○	○	○	○

If needed, please reformulate this competency

Before the response operation, plan for the storage and stockpiling of vaccines and prepare for medical and non-medical countermeasures.

	1	2	3	4	5	6	7	8	9
*	○	○	○	○	○	○	○	○	○

If needed, please reformulate this competency

Organization / Policy Development / Roles and Responsibilities

Identify key partners and develop a common understanding of roles, resources, planning assumptions, risks/vulnerabilities and information that should be shared during response operations.

	1	2	3	4	5	6	7	8	9
*	○	○	○	○	○	○	○	○	○

If needed, please reformulate this competency

Support the building of core capacities at designated Points of Entry (PoE) and understand the importance of supporting core capacity building.

	1	2	3	4	5	6	7	8	9
*	○	○	○	○	○	○	○	○	○

If needed, please reformulate this competency

Develop, test and evaluate a Public Health Emergency Contingency Plan (PHECP).

	1	2	3	4	5	6	7	8	9
*	○	○	○	○	○	○	○	○	○

If needed, please reformulate this competency

Provide healthcare workers with clinical quidelines for emerging infections from abroad, especially those that may be carried by travellers and the severely contagious.

	1	2	3	4	5	6	7	8	9
*	○	○	○	○	○	○	○	○	○

If needed, please reformulate this competency

Zijn er op het gebied van noodplanning (contingency planning) nog competenties die u hier mist? Denk bijvoorbeeld aan specifieke kennis, vaardigheden of houding die bij noodplanning belangrijk zijn? Schrijf hieronder uw suggesties op.

* = Invoer verplicht

<< Terug | Verder >> | Tussentijds bewaren

Rijksinstituut voor Volksgezondheid
en Milieu
Ministerie van Volksgezondheid,
Welzijn en Sport

Voortgangs balk

Surveillance

To what extent do you consider this competency as a relevant element for infectious disease preparedness at airports? On a 9-point Likert scale (1=totally irrelevant, 9 =totally relevant)

Medical / Public Health specific knowledge and skills

Recognize a potentially infectious disease by key symptoms and signs of events among travellers.

	1	2	3	4	5	6	7	8	9
*	○	○	○	○	○	○	○	○	○

If needed, please reformulate this competency

Understand the relevance for early detection of public health threats.

	1	2	3	4	5	6	7	8	9
*	○	○	○	○	○	○	○	○	○

If needed, please reformulate this competency

Understand the components of surveillance systems and how these work.

	1	2	3	4	5	6	7	8	9
*	○	○	○	○	○	○	○	○	○

If needed, please reformulate this competency

Interpret information from existing surveillance in order to characterize affected population groups, and to monitor disease trends and the impact of control strategies.

	1	2	3	4	5	6	7	8	9
*	○	○	○	○	○	○	○	○	○

If needed, please reformulate this competency

Use event-based and indicator-based surveillance systems to detect health threats.

	1	2	3	4	5	6	7	8	9
*	○	○	○	○	○	○	○	○	○

If needed, please reformulate this competency

Organization / Policy Development / Roles and Responsibilities

Understand the roles and responsibilities of local, national and international organizations involved in infectious disease control.

	1	2	3	4	5	6	7	8	9
*	○	○	○	○	○	○	○	○	○

If needed, please reformulate this competency

Be familiar with laws on surveillance and reporting of infectious diseases at national, EU level and globally (International Health Regulations)

	1	2	3	4	5	6	7	8	9
*	○	○	○	○	○	○	○	○	○

If needed, please reformulate this competency

Science

Conduct an epidemiological study, including writing a study protocol, conducting data management, reporting and presenting the results and recommending evidence-based interventions to decision makers.

	1	2	3	4	5	6	7	8	9
*	○	○	○	○	○	○	○	○	○

If needed, please reformulate this competency

Zijn er op het gebied van toezicht (surveillance) nog competenties die u hier mist? Denk bijvoorbeeld aan specifieke kennis, vaardigheden of houding die bij toezicht belangrijk zijn? Schrijf hieronder uw suggesties op.

* = Invoer verplicht

<< Terug | Verder >> | Tussentijds bewaren

Rijksinstituut voor Volksgezondheid en Milieu
Ministerie van Volksgezondheid, Welzijn en Sport

Voortgangs balk

Risk assessment

To what extent do you consider this competency as a relevant element for infectious disease preparedness at airports?
On a 9-point Likert scale (1=totally irrelevant, 9=totally relevant)

Medical / Public Health specific knowledge and skills

Understand risk analysis frameworks, with the elements of risk assessment, risk management and risk communication.

	1	2	3	4	5	6	7	8	9
*	○	○	○	○	○	○	○	○	○

If needed, please reformulate this competency

Determine when a risk assessment should be carried out and appropriate measures should be taken.

	1	2	3	4	5	6	7	8	9
*	○	○	○	○	○	○	○	○	○

If needed, please reformulate this competency

Perform a risk assessment and continuously review the risk assessment as further information becomes available.

	1	2	3	4	5	6	7	8	9
*	○	○	○	○	○	○	○	○	○

If needed, please reformulate this competency

Interpret the diagnostic and epidemiological significance of reports form laboratory tests.

	1	2	3	4	5	6	7	8	9
*	○	○	○	○	○	○	○	○	○

If needed, please reformulate this competency

Integrate and interpret information from a variety of local, national, and international sources regarding contaminants in air, soil and water.

	1	2	3	4	5	6	7	8	9
*	○	○	○	○	○	○	○	○	○

If needed, please reformulate this competency

Organization / Policy Development / Roles and Responsibilities

Collect and integrate the facts of an event, based on information from multiple sources including the traveller, the aircraft operator, gorund-based medical services for aircraft in flight (when available) or the agent responsible for the baggage or cargo.

	1	2	3	4	5	6	7	8	9
*	○	○	○	○	○	○	○	○	○

If needed, please reformulate this competency

Know when case reports or clusters require further investigation, and how to initiate such investigations.

	1	2	3	4	5	6	7	8	9
*	○	○	○	○	○	○	○	○	○

If needed, please reformulate this competency

Zijn er op het gebied van risicobeoordeling (risk assessment) nog competenties die u hier mist? Denk bijvoorbeeld aan specifieke kennis, vaardigheden of houding die bij risicobeoordeling belangrijk zijn? Schrijf hieronder uw suggesties op.

* = Invoer verplicht

<< Terug | Verder >> | Tussentijds bewaren

Rijksinstituut voor Volksgezondheid en Milieu
Ministerie van Volksgezondheid, Welzijn en Sport

Voortgangs balk

Outbreak investigation

To what extent do you consider this competency as a relevant element for infectious disease preparedness at airports? On a 9-point Likert scale (1=totally irrelevant, 9=totally relevant)

Medical / Public Health specific knowledge and skills

Conduct outbreak investigations to identify pathogens and other agents, characterize affected population groups, and sources of exposure .

	1	2	3	4	5	6	7	8	9
*	○	○	○	○	○	○	○	○	○

If needed, please reformulate this competency

Use reliable systems for disseminating case definitions to standardize both the diagnosis and the reporting of case numbers (e.g. confirmed, suspected, probable, or possible cases).

	1	2	3	4	5	6	7	8	9
*	○	○	○	○	○	○	○	○	○

If needed, please reformulate this competency

Systematically generate required information about the number of travellers such as those targeted for screening, screened, referred to secondary screening, and identified as confirmed cases.

	1	2	3	4	5	6	7	8	9
*	○	○	○	○	○	○	○	○	○

If needed, please reformulate this competency

Organization / Policy Development / Roles and Responsibilities

Identify who is responsible at the national level for receiving the information on the investigation from the local or intermediate level health authority.

	1	2	3	4	5	6	7	8	9
*	○	○	○	○	○	○	○	○	○

If needed, please reformulate this competency

Science

Have the biological, clinical and epidemiological knowledge needed to characterize (potentially) novel pathogens and other agents responsible for an outbreak disease.

	1	2	3	4	5	6	7	8	9
*	○	○	○	○	○	○	○	○	○

If needed, please reformulate this competency

Use evidence based methods to identify, and recommend control and preventive measures to control an outbreak.

	1	2	3	4	5	6	7	8	9
*	○	○	○	○	○	○	○	○	○

If needed, please reformulate this competency

Zijn er op het gebied van onderzoek naar uitbraken (outbreak investigation) nog competenties die u hier mist? Denk bijvoorbeeld aan specifieke kennis, vaardigheden of houding die bij onderzoek naar uitbraken belangrijk zijn? Schrijf hieronder uw suggesties op.

* = Invoer verplicht

<< Terug | Verder >> | Tussentijds bewaren

Rijksinstituut voor Volksgezondheid en Milieu
Ministerie van Volksgezondheid, Welzijn en Sport

Voortgangs balk

Response

U heeft de taken met bijbehorende competenties onder het **Preparedness** proces zojuist beoordeeld.

Nu gaat de vragenlijst verder met taken en competenties die specifiek toebehoren onder het **Response** proces.
Dat zijn; **Management of ill / exposed travellers** en **Public health measures**

* = Invoer verplicht

<< Terug | Verder >> | Tussentijds bewaren

Rijksinstituut voor Volksgezondheid en Milieu
Ministerie van Volksgezondheid, Welzijn en Sport

Voortgangs balk

Management of ill / exposed travellers

To what extent do you consider this competency as a relevant element for infectious disease preparedness at airports? On a 9-point Likert scale (1=totally irrelevant, 9 =totally relevant)

Medical / Public Health specific knowledge and skills

Provide ground-based medical support (GBMS) including medical recommendations during in-flight events and pre-flight passenger screenings for decisions regarding medical treatment and use of onboard medications or equipment.

	1	2	3	4	5	6	7	8	9
*	○	○	○	○	○	○	○	○	○

If needed, please reformulate this competency

Assess health status of travellers from an affected region or who have been exposed to a potential public health risk during air travel.

	1	2	3	4	5	6	7	8	9
*	○	○	○	○	○	○	○	○	○

If needed, please reformulate this competency

Prepare disembarking travellers with information on precautions to take in the event of illness, information sources for any updates on the event and public health authority (PHA) contact information for subsequent enquiries.

	1	2	3	4	5	6	7	8	9
*	○	○	○	○	○	○	○	○	○

If needed, please reformulate this competency

Organization / Policy Development / Roles and Responsibilities

Determine the appropriate parking stand for an incoming affected aircraft and the order of disembarkation of passengers.

	1	2	3	4	5	6	7	8	9
*	○	○	○	○	○	○	○	○	○

If needed, please reformulate this competency

Arrange efficient port health staff in order to reduce the time that travellers spend on a board-affected aircraft, and identify space requirements for interviews and health assessments of arriving travellers.

	1	2	3	4	5	6	7	8	9
*	○	○	○	○	○	○	○	○	○

If needed, please reformulate this competency

Arrange a possible transfer to a medical facility by ambulance and facilitate the rapid transport of suspected cases of an infectious disease.

	1	2	3	4	5	6	7	8	9
*	○	○	○	○	○	○	○	○	○

If needed, please reformulate this competency

Zijn er op het gebied van management van zieke/blootgestelde reizigers (management of ill / exposed travellers) nog competenties die u hier mist? Denk bijvoorbeeld aan specifieke kennis, vaardigheden of houding die bij management van zieke/blootgestelde reizigers belangrijk zijn? Schrijf hieronder uw suggesties op.

* = Invoer verplicht

<< Terug | Verder >> | Tussentijds bewaren

Voortgangs balk

Public health measures

To what extent do you consider this competency as a relevant element for infectious disease preparedness at airports? On a 9-point Likert scale (1=totally irrelevant, 9 =totally relevant)

Medical / Public Health specific knowledge and skills

Recognize when it is necessary to wear Personal Protective Equipment (PPE), which PPE is required, where the equipment is stored and how the PPE is donned or doffed.

	1	2	3	4	5	6	7	8	9
*	○	○	○	○	○	○	○	○	○

If needed, please reformulate this competency

Determine triggers for appropriate public health measures, such as travel restrictions, quarantine, treatment and isolation, that are commensurate with the risk and do not unduly interfere with international travel.

	1	2	3	4	5	6	7	8	9
*	○	○	○	○	○	○	○	○	○

If needed, please reformulate this competency

Relate information regarding medical clearance for travellers with health conditions that may affect their suitability for air travel.

	1	2	3	4	5	6	7	8	9
*	○	○	○	○	○	○	○	○	○

If needed, please reformulate this competency

Execute vaccination or other prophylaxis after the agreement of the traveller or his/her parents or quardians.

	1	2	3	4	5	6	7	8	9
*	○	○	○	○	○	○	○	○	○

If needed, please reformulate this competency

Determine, based on the results of the inspection. if further disinfection, decontamination, disinsection or derating measures of the aircraft of at the airport are required.

	1	2	3	4	5	6	7	8	9
*	○	○	○	○	○	○	○	○	○

If needed, please reformulate this competency

Recognize when to implement special handling of baggage or cargo from affected regions, including inspection, fumigation, and other decontamination of possibly destruction.

	1	2	3	4	5	6	7	8	9
*	○	○	○	○	○	○	○	○	○

If needed, please reformulate this competency

Organization / Policy Development / Roles and Responsibilities

Analyse the costs of the public health measures and resulting liabilities.

	1	2	3	4	5	6	7	8	9
*	○	○	○	○	○	○	○	○	○

If needed, please reformulate this competency

Prepare (aircraft) cleaning personnel, immigration personnel and cargo handlers with information regarding the public health event to protect themselves and healthy travellers as required.

	1	2	3	4	5	6	7	8	9
*	○	○	○	○	○	○	○	○	○

If needed, please reformulate this competency

Science

Arrange the use of public health measures by scientific evidence and expert public health opinions to avoid contradictory or unnecessary restrictions of individuals.

	1	2	3	4	5	6	7	8	9
*	○	○	○	○	○	○	○	○	○

If needed, please reformulate this competency

Zijn er op het gebied van volksgezondheidmaatregelen (public health measures) nog competenties die u hier mist? Denk bijvoorbeeld aan specifieke kennis, vaardigheden of houding die bij volksgezondheidmaatregelen belangrijk zijn? Schrijf hieronder uw suggesties op.

Rijksinstituut voor Volksgezondheid en Milieu
Ministerie van Volksgezondheid, Welzijn en Sport

Voortgangs balk

Recovery

U heeft de taken met bijbehorende competenties onder het **Response** proces zojuist beoordeeld.

De vragenlijst sluit af met competenties die specifiek toebehoren onder het **Recovery** proces.
Dat is: **evaluation and recovery**

* = Invoer verplicht

<< Terug **Verder >>** **Tussentijds bewaren**

Rijksinstituut voor Volksgezondheid
en Milieu
Ministerie van Volksgezondheid,
Welzijn en Sport

Voortgangs balk

Evaluation and recovery

To what extent do you consider this competency as a relevant element for infectious disease preparedness at airports? On a 9-point Likert scale (1=totally irrelevant, 9 =totally relevant)

Medical / Public Health specific knowledge and skills

Clearly define goals and objectives of the evaluation.

	1	2	3	4	5	6	7	8	9
*	○	○	○	○	○	○	○	○	○

If needed, please reformulate this competency

Develop a formal evaluation of the response and share with all stakeholders, when the public health is under control or concluded.

	1	2	3	4	5	6	7	8	9
*	○	○	○	○	○	○	○	○	○

If needed, please reformulate this competency

Organization / Policy Development / Roles and Responsibilities

Deactivate the plan and return to recovery once the situation is under control or able to be de-escalated.

	1	2	3	4	5	6	7	8	9
*	○	○	○	○	○	○	○	○	○

If needed, please reformulate this competency

Plan for the demobilization and recovery of healthcare workers after a response operation.

	1	2	3	4	5	6	7	8	9
*	○	○	○	○	○	○	○	○	○

If needed, please reformulate this competency

Update plans according the key lessons learnt after a formal review.

	1	2	3	4	5	6	7	8	9
*	○	○	○	○	○	○	○	○	○

If needed, please reformulate this competency

In vergelijking met de hoeveelheid competenties voor preparedness (voorbereiding) en response (reactie), is de hoeveelheid voor recovery (herstel) vrij klein. Er is minder over recovery in de literatuur beschreven. **Zijn er op het gebied van evaluatie en herstel nog competenties die u hier mist? Schrijf hieronder uw suggesties op. Denk hierbij o.a. aan aspecten van communicatie, samenwerking, professionaliteit, publieke gezondheid / medisch inhoudelijke kennis, organisatie en wetenschap.**

Rijksinstituut voor Volksgezondheid
en Milieu
Ministerie van Volksgezondheid,
Welzijn en Sport

Voortgangs balk

Slotvraag

Is dit competentieprofiel een afspiegeling van uw houding, kennis en vaardigheden bij infectieziekte voorbereiding en bestrijding op Schiphol?

- Ja
- Gedeeltelijk
- Nee

Indien u gedeeltelijk of niet hebt geantwoord, welke onderwerpen mist u? Geef een omschrijving van deze onderwepen met bijbehorende kennis, vaardigheden en houding. Indien mogelijk formuleer competenties aan de hand van het formuleer advies in de bijlage.

* = Invoer verplicht

<< Terug | Verder >> | Tussentijds bewaren

Rijksinstituut voor Volksgezondheid
en Milieu
Ministerie van Volksgezondheid,
Welzijn en Sport

Voortgangs balk

Hartelijk dank voor het invullen van de vragenlijst.
Vergeet niet onderaan op 'verzenden' te klikken.

Uw persoonlijk feedback rapport ontvangt u in de week van 10 tot 14 juni. Indien u deelneemt aan de groepsdiscussie zien wij u graag donderdag 20 juni. De officiële uitnodiging voor de groepsdiscussie ontvangt u spoedig.

Hartelijke groet,

Namens het onderzoeksteam,
Prof. Dr. Aura Timen
Dr. Evelien Belfroid
Doret de Rooij, MD
Rebekka Rebel [redacted]

* = Invoer verplicht

<< Terug | Tussentijds bewaren | Verzenden
