# Supplementary material for: Development of a competency profile for professionals involved in infectious disease preparedness and response in the air transport public health sector
Source: PLoS One. 2020 May 21;15(5):e0233360. doi: 10.1371/journal.pone.0233360 (PMC7241746; doi:10.1371/journal.pone.0233360)
Supplement: S2 Questionnaire — (PDF) [file pone.0233360.s006.pdf]

S6. Questionnaire International Data Collection

Rijksinstituut voor Volksgezondheid
en Milieu
Ministerie van Volksgezondheid,
Welzijn en Sport

Progress bar

A competency profile for professionals involved in infectious disease control at designated airports.

Introduction
You recently received an invitation to participate in this study. We developed a competency profile for the interdisciplinary group of airport professionals at one major airport, and now aim to internationally validate this profile among international professionals. This study is conducted by the National Insitute for Public Health and the Environment (RIMV) and is part of a PhD trajectory on strenghtening the preparation and response capacity at ports, airports and ground-crossings (points-of-entry (POE)).

Background
Infectious diseases are able to spread globally through air travel. Although events might occur irregularly, regular training or exercising these scenarios is a good way to remain prepared. To design effective training or exercises, it is important to know what professionals at airports actually have to know and do; it is important to know which competencies are required. Although general competency profiles in infectious disease control exist, there is no competency profile available which is tailored to the specific airport tasks and settings.

Why do you receive this questionnaire?
Your expertise as a professional at one of Europe's major airports is mostly wanted to develop a practical and useful profile. We ask you to rate the relevance of the competencies in this questionnaire and provide feedback on formulations or additional competencies.
When filling in the questionnaire, it is important that you keep in mind that these competencies need to apply specifically to airports.
Therefore, we ask you to:

- Assess competencies on relevancy for infectious disease control at airports.
- Provide feedback on formulations or additional competencies using the attached guide on how to formulate competencies. We recommend that you print this quide out and keep it next to you while filling in the questionnaire.

Structure of the competency profile
The profile with a total of 60 competencies consists out of three processes: (**Preparedness**), (**Response**) and (**Recovery**) with associated tasks. These tasks are subcategorized in: Medical / Public health - specific knowledge and skills, Organization / Policy development / Roles and responsibilities and Science.

Competenties regarding **Communication, Collaboration** en **Professionalism** apply to all processes.

Completing the questionnaire takes approximately 20 to 30 minutes.

Consensus meeting
Competencies which are scored alternately relevant will be discussed during a 2-hour meeting where participants of the questionnaire will be present. This will take place during the trainer-of-trainer meeting in Belgrade, Serbia, between 18-20 September 2019.
You will receive an official invitation for and additional information about this meeting soon. Furthermore, you will receive and personal feedback report in advance of the consensus meeting. In this report, you will find the results of the questionnaire by means of both your own answers next to the average score of the group.

Do you have questions about the questionnaire or this study?
If you have any problems or questions, please do not hesitate to contact us via [redacted]

* = Input is required

Next >> | Store entries, finish later

Rijksinstituut voor Volksgezondheid en Milieu
Ministerie van Volksgezondheid, Welzijn en Sport

Progress bar

Informed consent

Before, during and after the study, your personal data will be handled carefully and confidentially. The results of this study will be published in general terms and will never be reducible to an individual. Participation in this study is voluntary and withdrawal from the study is possible at any time.

By clicking on the icon below you agree to the collection and use of some personal data.
This concerns data such as your gender, profession, and the number of years working in your current profession.
This information is stored for 15 years, but are only accessible to the involved RIVM scientists.
You can read more about this in the RIVM privacy statement:https://www.rivm.nl/sites/default/files/2018-11/RIVM%20privacyverklaring%20mei%202018%20definitief%20Engels.pdf
*

○ Yes, I agree

* = Input is required

<< Back | Next >> | Store entries, finish later

Rijksinstituut voor Volksgezondheid en Milieu
Ministerie van Volksgezondheid, Welzijn en Sport

Progress bar

Guidance on how to formulate competencies

Before you start with the questionnaire, we will give you a brief instruction on how to formulate competencies.
Competencies have two or three parts;

1. A verb to specify the cognitive level at which you expect the professionals to perform
2. The object/topic professionals' knowledge, skill. attitude relates to

Recommended:
3. An adverb to define the quality of the action

Examples

- Recognize a potentially infectious disease by key symptoms and signs of events among travellers.
- Rapidly facilitate the transport of suspected cases of an infectious disease.

* = Input is required

<< Back | Next >> | Store entries, finish later

Rijksinstituut voor Volksgezondheid
en Milieu
Ministerie van Volksgezondheid,
Welzijn en Sport

Progress bar

The questionnaire starts on the next page. Try to fill in the questionnaire as completely as possible.

We kindly ask you to complete the questionnaire before the 4st of September.

* = Input is required

<< Back | Next >> | Store entries, finish later

Rijksinstituut voor Volksgezondheid
en Milieu
Ministerie van Volksgezondheid,
Welzijn en Sport

Progress bar

Demographic questions

I'm a

- ○ Man
- ○ Woman

What is your current profession in relation to infectious disease control at the airport?

How many years are you working in your current profession?

Which of the following partners do you collaborate with the most?

- Public health authority
- Airport Medical Services
- Airlines

Other, namely;

To what extent do you feel experienced with infectious disease control at airports?

- ○ 1 (very inexperienced)
- ○ 2 (inexperienced)
- ○ 3 (neither experienced/inexperienced)
- ○ 4 (experienced)
- ○ 5 (very experienced)

Please, enter the e-mail address through which we invited you below.

If you prefer to receive the personal feedback report through another e-mail address, please enter it below.

* = Input is required

<< Back | Next >> | Store entries, finish later

Rijksinstituut voor Volksgezondheid en Milieu
Ministerie van Volksgezondheid, Welzijn en Sport

Progress bar

You have just answered the demographic questions. The questionnaire now continues with general competencies regarding **Communication** (n=2), **Collaboration** (n=3) & **Professionalism** (n=2).

Competencies might feel quite theoretic. For your own convenience, we suggest that you imagine a case of an infectious disease event that could happen at your airport while you rate the competencies.

* = Input is required

<< Back | Next >> | Store entries, finish later

Rijksinstituut voor Volksgezondheid en Milieu
Ministerie van Volksgezondheid, Welzijn en Sport

Progress bar

Communication

To what extent do you consider this competency as a relevant element for infectious disease control at airports? On a 9-point Likert scale (1=totally irrelevant, 9 =totally relevant)

Understand and implement the basic principles of risk communication to airport and airline staff, travellers, the public and media.

	1	2	3	4	5	6	7	8	9
*	○	○	○	○	○	○	○	○	○

If needed, please reformulate this competency

Establish trust with healthcare providers through rapid communication channels and ongoing two-way communication.

	1	2	3	4	5	6	7	8	9
*	○	○	○	○	○	○	○	○	○

If needed, please reformulate this competency

Do you still miss any competencies on communication? Consider for example specific knowledge, skills or attitudes towards communication. Please, write down your suggestions below.

* = Input is required

<< Back | Next >> | Store entries, finish later

Rijksinstituut voor Volksgezondheid en Milieu
Ministerie van Volksgezondheid, Welzijn en Sport

Progress bar

Collaboration

To what extent do you consider this competency as a relevant element for infectious disease control at airports? On a 9-point Likert scale (1=totally irrelevant, 9 =totally relevant)

Understand the importance of multidisciplinary collaboration during acute outbreak management.

	1	2	3	4	5	6	7	8	9
*	○	○	○	○	○	○	○	○	○

If needed, please reformulate this competency

Be an effective team member, adopting the role needed to contribute constructively to the accomplishment of tasks by the group.

	1	2	3	4	5	6	7	8	9
*	○	○	○	○	○	○	○	○	○

If needed, please reformulate this competency

Participate in the implementation of established plans, which ensure continuity of operations.

	1	2	3	4	5	6	7	8	9
*	○	○	○	○	○	○	○	○	○

If needed, please reformulate this competency

Do you still miss any competencies on collaboration? Consider for example specific knowledge, skills and attitudes towards collaboration? Please, write down your suggestions below.

* = Input is required

<< Back | Next >> | Store entries, finish later

Rijksinstituut voor Volksgezondheid
en Milieu
Ministerie van Volksgezondheid,
Welzijn en Sport

Progress bar

Professionalism

Minimize crewmembers', ground staff', and passengers ' any discomfort or distress associated with public health measures.

	1	2	3	4	5	6	7	8	9
*	○	○	○	○	○	○	○	○	○

If needed, please reformulate this competency

Do you still miss any competencies on professionalism? Consider for example recent hot topics such as data sharing in light of the GDPR, or other specific knowledge, skills or attitudes towards professionalism? Please, write down your suggestions below.

* = Input is required

<< Back | Next >> | Store entries, finish later

Rijksinstituut voor Volksgezondheid
en Milieu
Ministerie van Volksgezondheid,
Welzijn en Sport

Progress bar

Preparedness

You have just assessed the general competencies regarding Communication, Collaboration and Professionalism.

The questionnaire now continues with tasks that specifically belong to **Preparedness.**
These are: **Training** (n=3), **Contingency planning** (n=9) and **Surveillance** (n=7)

* = Input is required

<< Back | Next >> | Store entries, finish later

Rijksinstituut voor Volksgezondheid
en Milieu
Ministerie van Volksgezondheid,
Welzijn en Sport

Progress bar

Training

To what extent do you consider this competency as a relevant element for infectious disease control at airports? On a 9-point Likert scale (1=totally irrelevant, 9 =totally relevant)

Medical / Public Health - specific knowledge and skills

Provide training, include healthcare providers in training and exercise on required communication skills.

	1	2	3	4	5	6	7	8	9
*	○	○	○	○	○	○	○	○	○

If needed, please reformulate this competency

Organization / Policy development / Roles and Responsibilities

Identify training needs, planning and organizing courses.

	1	2	3	4	5	6	7	8	9
*	○	○	○	○	○	○	○	○	○

If needed, please reformulate this competency

Periodically practice and test the ability to make decisions under uncertainty

	1	2	3	4	5	6	7	8	9
*	○	○	○	○	○	○	○	○	○

If needed, please reformulate this competency

Do you still miss any competencies on training? Consider for example specific knowledge, skills or attitudes towards training? Please, write down your suggestions below.

* = Input is required

<< Back | Next >> | Store entries, finish later

Rijksinstituut voor Volksgezondheid en Milieu
Ministerie van Volksgezondheid, Welzijn en Sport

Progress bar

Contingency planning

To what extent do you consider this competency as a relevant element for infectious disease control at airports? On a 9-point Likert scale (1= totally relevant, 9=totally relevant)

Medical / Public Health specific knowledge and skills

Be familiar with job-related standards and recommended practices concerning infectious disease control from national and international aviation organizations (IATA, ICAO and CAPSCA).

	1	2	3	4			5		
*	○	○	○	○	○	○	○	○	○

If needed, please reformulate this competency

Periodically assess if the implementation of strategies, standard operationg procedures (SOPs) and action plans requires any changes.

	1	2	3	4	5	6	7	8	9
*	○	○	○	○	○	○	○	○	○

If needed, please reformulate this competency

Before the response operation, identify which triggers will require key decisions during outbreak response (keeping in mind that triggers may need modification to fit specific situations).

	1	2	3	4	5	6	7	8	9
*	○	○	○	○	○	○	○	○	○

If needed, please reformulate this competency

Before the response operation, plan for the storage and stockpiling of medical and non-medical countermeasures.

	1	2	3	4	5	6	7	8	9
*	○	○	○	○	○	○	○	○	○

If needed, please reformulate this competency

Organization / Policy Development / Roles and Responsibilities

Understand the local and logistical structure of the airport and the international context of airports and their functioning.

	1	2	3	4			5		
*	○	○	○	○	○	○	○	○	○

If needed, please reformulate this competency

Identify key partners and develop a common understanding of roles, resources, planning assumptions, risks/vulnerabilities and information needs.

	1	2	3	4	5	6	7	8	9
*	○	○	○	○	○	○	○	○	○

If needed, please reformulate this competency

Support the building of core capacities at the airport and understand the importance of supporting core capacity building.

	1	2	3	4	5	6	7	8	9
*	○	○	○	○	○	○	○	○	○

If needed, please reformulate this competency

Develop, test and evaluate a Public Health Emergency Contingency Plan (PHECP) on a periodical basis.

	1	2	3	4	5	6	7	8	9
*	○	○	○	○	○	○	○	○	○

If needed, please reformulate this competency

Provide relevant airport and airline staff with quidelines regarding emerging infections from abroad, especially those that may be carried by travellers and the severely contagious.

	1	2	3	4	5	6	7	8	9
*	○	○	○	○	○	○	○	○	○

If needed, please reformulate this competency

Do you still miss any competencies on contingency planning? Consider for example specific knowledge, skills or attitudes towards contingency planning? Please, write down your suggestions below.

* = Input is required

<< Back | Next >> | Store entries, finish later

Rijksinstituut voor Volksgezondheid
en Milieu
*Ministerie van Volksgezondheid,
Welzijn en Sport*

Progress bar

Surveillance

To what extent do you consider this competency as a relevant element for infectious disease control at airports? On a 9-point Likert scale (1=totally irrelevant, 9 =totally relevant)

Medical / Public Health specific knowledge and skills

Recognize a potentially infectious disease by key symptoms and signs of events among travellers.

	1	2	3	4	5	6	7	8	9
*	○	○	○	○	○	○	○	○	○

If needed, please reformulate this competency

Understand the relevance of early detection of public health threats.

	1	2	3	4	5	6	7	8	9
*	○	○	○	○	○	○	○	○	○

If needed, please reformulate this competency

Understand the components of surveillance systems and how these work.

	1	2	3	4	5	6	7	8	9
*	○	○	○	○	○	○	○	○	○

If needed, please reformulate this competency

Interpret information from existing surveillance in order to characterize affected population groups, and to monitor disease trends and the impact of control strategies.

	1	2	3	4	5	6	7	8	9
*	○	○	○	○	○	○	○	○	○

If needed, please reformulate this competency

Use event-based and indicator-based surveillance systems to detect health threats.

	1	2	3	4	5	6	7	8	9
*	○	○	○	○	○	○	○	○	○

If needed, please reformulate this competency

Organization / Policy Development / Roles and Responsibilities

Understand the roles and responsibilities of local, national and international organizations involved in infectious disease control.

	1	2	3	4	5	6	7	8	9
*	○	○	○	○	○	○	○	○	○

If needed, please reformulate this competency

Be familiar with laws on surveillance and reporting of infectious diseases at national, EU level and globally (International Health Regulations)

	1	2	3	4	5	6	7	8	9
*	○	○	○	○	○	○	○	○	○

If needed, please reformulate this competency

Do you still miss any competencies on surveillance? Consider for example specific knowledge, skills or attitudes towards surveillance? Please, write down your suggestions below.

* = Input is required

<< Back **Next >>** **Store entries, finish later**

Rijksinstituut voor Volksgezondheid
en Milieu
Ministerie van Volksgezondheid,
Welzijn en Sport

Progress bar

Response

You have just assessed the tasks with assosicated competencies belonging to **Preparedness.**

The questionnaire now continues with tasks that specifically belong to **Response**.
These are: **Risk assessment** (n=7), **Outbreak investigations** (n=8), **Management of ill / exposed travellers** (n=6) and **Public health measures** (9).

* = Input is required

<< Back **Next >>** **Store entries, finish later**

Progress bar

Risk assessment

**To what extent do you consider this competency as a relevant element for infectious disease control at airports?
On a 9-point Likert scale (1=totally irrelevant, 9=totally relevant)**

Medical / Public Health specific knowledge and skills

Understand risk analysis frameworks, with the elements of risk assessment, risk management and risk communication.

	1	2	3	4	5	6	7	8	9
*	○	○	○	○	○	○	○	○	○

If needed, please reformulate this competency

Determine when a risk assessment should be carried out and appropriate measures should be taken.

	1	2	3	4	5	6	7	8	9
*	○	○	○	○	○	○	○	○	○

If needed, please reformulate this competency

Perform a risk assessment and continuously review the risk assessment as further information becomes available.

	1	2	3	4	5	6	7	8	9
*	○	○	○	○	○	○	○	○	○

If needed, please reformulate this competency

Interpret the diagnostic and epidemiological significance of reports form laboratory tests.

	1	2	3	4	5	6	7	8	9
*	○	○	○	○	○	○	○	○	○

If needed, please reformulate this competency

Integrate and interpret information from a variety of local, national, and international sources regarding contaminants in air, soil and water.

	1	2	3	4	5	6	7	8	9
*	○	○	○	○	○	○	○	○	○

If needed, please reformulate this competency

Organization / Policy Development / Roles and Responsibilities

Collect and integrate the facts of an event, based on information from multiple sources including the traveller, the aircraft operator, ground-based medical services for aircraft in flight (when available) or the agent responsible for the baggage or cargo.

	1	2	3	4	5	6	7	8	9
*	○	○	○	○	○	○	○	○	○

If needed, please reformulate this competency

Know when case reports or clusters require further investigation, and how to initiate such investigations.

	1	2	3	4	5	6	7	8	9
*	○	○	○	○	○	○	○	○	○

If needed, please reformulate this competency

Do you still miss any competencies on risk assessment? Consider for example specific knowledge, skills or attitudes towards risk assessment. Please, write down your suggestions below.

* = Input is required

<< Back | Next >> | Store entries, finish later

Rijksinstituut voor Volksgezondheid en Milieu
Ministerie van Volksgezondheid, Welzijn en Sport

Progress bar

Outbreak investigation

To what extent do you consider this competency as a relevant element for infectious disease control at airports? On a 9-point Likert scale (1=totally irrelevant, 9=totally relevant)

Medical / Public Health specific knowledge and skills

Conduct outbreak investigations to identify pathogens and other agents, characterize affected population groups, and sources of exposure .

	1	2	3	4	5	6	7	8	9
*	○	○	○	○	○	○	○	○	○

If needed, please reformulate this competency

Use reliable systems for disseminating case definitions to standardize both the diagnosis and the reporting of case numbers (e.g. confirmed, suspected, probable, or possible cases).

	1	2	3	4	5	6	7	8	9
*	○	○	○	○	○	○	○	○	○

If needed, please reformulate this competency

Systematically generate required information about the number of travellers such as those targeted for screening, screened, referred to secondary screening, and identified as confirmed cases.

	1	2	3	4	5	6	7	8	9
*	○	○	○	○	○	○	○	○	○

If needed, please reformulate this competency

Implement contact tracing based on a careful, case-by-case, risk assessment with taking into account factors such as feasibility, the severity of the disease and its potential for epidemic spread, the infectivity of index patients, and the duration of the trip.

	1	2	3	4	5	6	7	8	9
	○	○	○	○	○	○	○	○	○

If needed, please reformulate this competency

Organization / Policy Development / Roles and Responsibilities

Identify who is responsible at the national level for receiving the information on the investigation from the local or intermediate level health authority.

	1	2	3	4	5	6	7	8	9
*	○	○	○	○	○	○	○	○	○

If needed, please reformulate this competency

Science

Maintain up-to-date and job-specific knowledge about characteristics of infectious diseases such as reservoir, potential sources, modes of transmission, risk groups and duration.

	1	2	3	4	5	6	7	8	9
*	○	○	○	○	○	○	○	○	○

If needed, please reformulate this competency

Be able to contact professionals that have the biological, clinical, and epidemiological knowledge needed to characterize (potentially novel) pathogens and other agents responsible for an outbreak disease.

	1	2	3	4	5	6	7	8	9
	○	○	○	○	○	○	○	○	○

If needed, please reformulate this competency

Use evidence based methods to identify, and recommend control and preventive measures to control an outbreak.

	1	2	3	4	5	6	7	8	9
*	○	○	○	○	○	○	○	○	○

If needed, please reformulate this competency

Do you still miss any competencies on outbreak investigations? Consider for example specific knowledge, skills and attitudes towards outbreak investigations? Please, write down your suggestions below.

* = Input is required

<< Back | Next >> | Store entries, finish later

Rijksinstituut voor Volksgezondheid
en Milieu
Ministerie van Volksgezondheid,
Welzijn en Sport

Progress bar

Management of ill / exposed travellers

To what extent do you consider this competency as a relevant element for infectious disease control at airports?
On a 9-point Likert scale (1=totally irrelevant, 9=totally relevant)

Medical / Public Health specific knowledge and skills

Provide ground-based medical support (GBMS) regarding infectious disease events, including medical recommendations to manage a suspected communicable disease during flights for decisions regarding medical treatment and use of onboard medications or equipment.

	1	2	3	4	5	6	7	8	9
*	○	○	○	○	○	○	○	○	○

If needed, please reformulate this competency

Assess travellers' health status and travel history from or going to an affected region or who have been exposed to a potential public health risk during air travel.

	1	2	3	4	5	6	7	8	9
*	○	○	○	○	○	○	○	○	○

If needed, please reformulate this competency

Prepare disembarking travellers with information on precautions to take in the event of illness, information sources for any updates on the event and public health authority (PHA) contact information for subsequent enquiries.

	1	2	3	4	5	6	7	8	9
*	○	○	○	○	○	○	○	○	○

If needed, please reformulate this competency

Organization / Policy Development / Roles and Responsibilities

Provide advice concerning the appropriate parking stand for an incoming affected aircraft and the order of disembarkation of passengers.

	1	2	3	4	5	6	7	8	9
*	○	○	○	○	○	○	○	○	○

If needed, please reformulate this competency

Provide advice concerning efficient port health staff in order to reduce the time that travellers spend on a board-affected aircraft, and identify space requirements for interviews and health assessments of arriving travellers.

	1	2	3	4	5	6	7	8	9
*	○	○	○	○	○	○	○	○	○

If needed, please reformulate this competency

Provide advice on a travellers's possible transfer to a medical facility by ambulance and facilitate the rapid transport of suspected cases of an infectious disease.

	1	2	3	4	5	6	7	8	9
*	○	○	○	○	○	○	○	○	○

If needed, please reformulate this competency

Do you still miss any competencies on management of ill / exposed travellers? Consider for example specific knowledge, skills or attitudes towards management of ill / exposed travellers. Please, write down your suggestions below.

* = Input is required

<< Back | Next >> | Store entries, finish later

Rijksinstituut voor Volksgezondheid
en Milieu
Ministerie van Volksgezondheid, Welzijn en Sport

Progress bar

Public health measures

To what extent do you consider this competency as a relevant element for infectious disease control at airports? On a 9-point Likert scale (1=totally irrelevant, 9 =totally relevant)

Medical / Public Health specific knowledge and skills

Recognize when it is necessary to wear Personal Protective Equipment (PPE), which PPE is required, where the equipment is stored and how the PPE is donned or doffed.

	1	2	3	4	5	6	7	8	9
*	○	○	○	○	○	○	○	○	○

If needed, please reformulate this competency

Determine triggers for appropriate public health measures, such as travel restrictions, quarantine, treatment and isolation, that are commensurate with the risk and do not unduly interfere with international travel.

	1	2	3	4	5	6	7	8	9
*	○	○	○	○	○	○	○	○	○

If needed, please reformulate this competency

Relate information regarding medical clearance for travellers with health conditions that may affect their suitability for air travel.

	1	2	3	4	5	6	7	8	9
*	○	○	○	○	○	○	○	○	○

If needed, please reformulate this competency

Provide information regarding vaccination or other prophylaxis after the agreement of the traveller or his/her parents or quardians.

	1	2	3	4	5	6	7	8	9
*	○	○	○	○	○	○	○	○	○

If needed, please reformulate this competency

Determine, based on the results of the inspection. if further disinfection, decontamination, disinsection or derating measures of the aircraft of at the airport are required.

	1	2	3	4	5	6	7	8	9
*	○	○	○	○	○	○	○	○	○

If needed, please reformulate this competency

Recognize when to implement special handling of baggage or cargo from affected regions, including inspection, fumigation, and other decontamination of possibly destruction.

	1	2	3	4	5	6	7	8	9
*	○	○	○	○	○	○	○	○	○

If needed, please reformulate this competency

Organization / Policy Development / Roles and Responsibilities

Assess whether the costs of the public health measures and resulting liabilities are proportionate to the risk.

	1	2	3	4	5	6	7	8	9
*	○	○	○	○	○	○	○	○	○

If needed, please reformulate this competency

Prepare relevant airport and airline staff with information regarding the public health event to protect themselves and healthy travellers as required.

	1	2	3	4	5	6	7	8	9
*	○	○	○	○	○	○	○	○	○

If needed, please reformulate this competency

Science

Arrange the use of public health measures by scientific evidence and expert public health opinions to avoid contradictory or unnecessary restrictions of individuals.

	1	2	3	4	5	6	7	8	9
*	○	○	○	○	○	○	○	○	○

If needed, please reformulate this competency

Do you still miss any competencies on public health measures? Consider for example specific knowledge, skills and attitudes towards public health measures? Please, write down your suggestions.

Rijksinstituut voor Volksgezondheid
en Milieu
*Ministerie van Volksgezondheid,
Welzijn en Sport*

Progress bar

Recovery

You have just assessed the tasks with associated competencies beloging to **Response.**

The questionnaire now ends with a task that specifically belong to **Recovery**.
That is: **Evaluation and recovery** (n=4).

* = Input is required

<< Back | Next >> | Store entries, finish later

Rijksinstituut voor Volksgezondheid
en Milieu
*Ministerie van Volksgezondheid,
Welzijn en Sport*

Progress bar

Evaluation and recovery

To what extent do you consider this competency as a relevant element for infectious disease control at airports? On a 9-point Likert scale (1=totally irrelevant, 9 =totally relevant)

Medical / Public Health specific knowledge and skills

Clearly define goals and objectives of the evaluation.

	1	2	3	4	5	6	7	8	9
*	○	○	○	○	○	○	○	○	○

If needed, please reformulate this competency

Develop a formal evaluation of the response and share with all stakeholders, when the public health is under control or concluded.

	1	2	3	4	5	6	7	8	9
*	○	○	○	○	○	○	○	○	○

If needed, please reformulate this competency

Organization / Policy Development / Roles and Responsibilities

Deactivate the plan and return to recovery once the situation is under control or able to be de-escalated.

	1	2	3	4	5	6	7	8	9
*	○	○	○	○	○	○	○	○	○

If needed, please reformulate this competency

Update plans according the key lessons learnt after a formal review.

	1	2	3	4	5	6	7	8	9
*	○	○	○	○	○	○	○	○	○

If needed, please reformulate this competency

The amount of competencies for recovery is relatively small compared to preparedness and response. Less has been described about recovery in the literature. Are there any competencies that you are missing here regarding recovery? Think of aspects such as communication, collaboration, professionalism, public health / medical knowledge, organization and science. Please, write down your suggestions below.

* = Input is required

<< Back | Next >> | Store entries, finish later

Rijksinstituut voor Volksgezondheid en Milieu
Ministerie van Volksgezondheid, Welzijn en Sport

Progress bar

Final questions

Now that you have assessed the competency profile, do you consider this profile as a reflection of your knowledge, skills and attitudes regarding infectious disease control at the airport?

- Yes
- Partially
- No

If you answered 'partially' or 'no', which competencies are you missing in this profile? If possible, please formulate competencies using the guidance in the attachments.

If you imagined any infectious disease event during this questionnaire, which specific event(s) did you use?

* = Input is required

<< Back | Next >> | Store entries, finish later

Rijksinstituut voor Volksgezondheid en Milieu
Ministerie van Volksgezondheid, Welzijn en Sport

Progress bar

Thank you for filling in the questionnaire.
Don't forget to click on the 'send' bottom below.

You will receive your personal feedback report between 11 and 17 September.

Yours sincerely,

On behalf of the research team,

Prof. Dr. Aura Timen, MD
Drs. Doret de Rooij, MD [redacted]
Rebekka Rebel [redacted]

* = Input is required

<< Back | Store entries, finish later | Send
